# Sensitive identification of neoantigens and cognate TCRs in human solid tumors

**DOI:** 10.1038/s41587-021-01072-6

**Published:** 2021-11-15

**Authors:** Marion Arnaud, Johanna Chiffelle, Raphael Genolet, Blanca Navarro Rodrigo, Marta A. S. Perez, Florian Huber, Morgane Magnin, Tu Nguyen-Ngoc, Philippe Guillaume, Petra Baumgaertner, Chloe Chong, Brian J. Stevenson, David Gfeller, Melita Irving, Daniel E. Speiser, Julien Schmidt, Vincent Zoete, Lana E. Kandalaft, Michal Bassani-Sternberg, Sara Bobisse, George Coukos, Alexandre Harari

**Affiliations:** 1 Ludwig Institute for Cancer Research, Lausanne Branch - University of Lausanne (UNIL), Lausanne, Switzerland; 2 Centre des Thérapies Expérimentales (CTE), Department of Oncology - Centre Hospitalier Universitaire Vaudois (CHUV), Lausanne, Switzerland; 3 Department of Oncology - University of Lausanne (UNIL) and Lausanne University Hospital (CHUV), Lausanne, Switzerland; 4 SIB Swiss Institute of Bioinformatics, Lausanne, Switzerland

**Keywords:** T-cell receptor, Cancer immunotherapy, Translational research

## Abstract

The identification of patient-specific tumor antigens is complicated by the low frequency of T cells specific for each tumor antigen. Here we describe NeoScreen, a method that enables the sensitive identification of rare tumor (neo)antigens and of cognate T cell receptors (TCRs) expressed by tumor-infiltrating lymphocytes. T cells transduced with tumor antigen-specific TCRs identified by NeoScreen mediate regression of established tumors in patient-derived xenograft mice.

## Abstract (web summary)

NeoScreen helps identify rare tumor antigens for personalized cancer vaccines and T cell therapies.

## Main

Cancer immunotherapies based on therapeutic vaccination or on the transfer of tumor-infiltrating lymphocytes (TILs) targeting tumor neoantigens have shown promising clinical outcomes^1–5^. Furthermore, engineering of blood T cells with tumor-reactive TCRs further expanded the horizons of adoptive T cell therapy (ACT)^6–10^. Identification of clinically relevant tumor antigens and their cognate TCRs^11–14^ is a critical foundation for such therapies. To this end, in vitro expanded autologous TILs^1,3,12,15–20^ and/or peripheral blood lymphocytes (PBLs)^4,14,15,21–26^ are usually interrogated for tumor antigen discovery. However, the frequency of neoantigen-specific T cells in PBLs and TILs is often low^15,22,23,26,27^, and we and others have shown that PBL and TIL repertoires are discordant^15,22,23,26^. Also, antigen discovery in PBLs remains challenging, despite pioneer work^14^ improving the detection of neoantigen reactivity in blood. Although use of TILs could be advantageous^15^, traditional culture methods for in vitro TIL expansion have been shown to skew the ex vivo TIL repertoire^28^, thus likely underestimating the quantification of tumor-reactive lymphocytes and curtailing the validation of tumor epitopes.

**Fig. 1:**
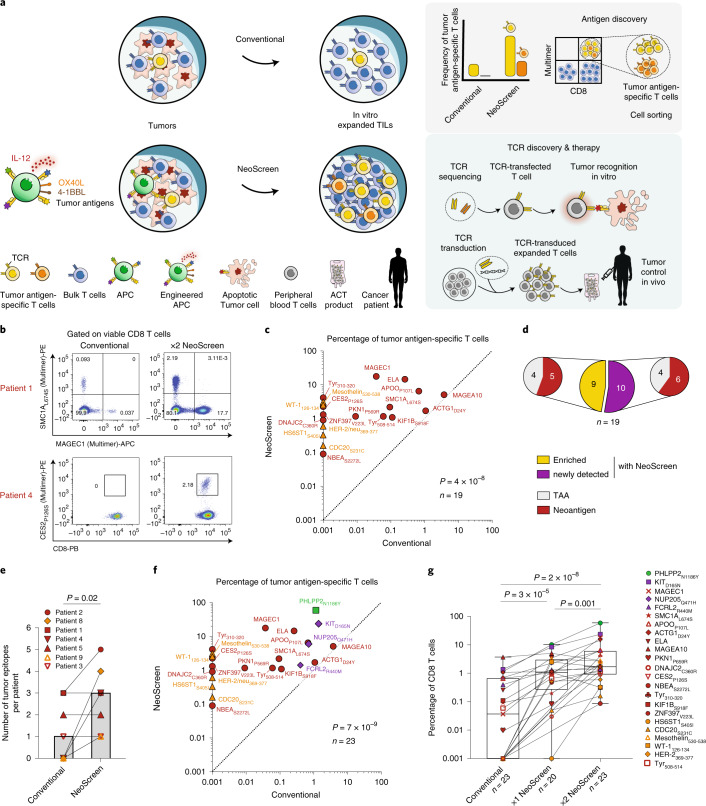
Sensitive tumor antigen discovery. **a**, NeoScreen pipeline. **b**–**e**, Antigen discovery with NeoScreen (*n* *=* 7 patients). **b**,**c**, Representative examples of flow cytometry data (**b**) and cumulative frequencies (**c**) of tumor antigen-specific CD8 T cells (*n* *=* 19 epitopes) in conventional (*x* axis) and NeoScreen (*y* axis) TIL cultures, by pMHC-multimers or 4-1BB upregulation. **d**, Proportions of neoepitope- versus TAA-specific among enriched versus newly detected T cell reactivities. **e**, Number of tumor epitopes per patient identified with conventional and NeoScreen strategies (histograms report median values). **f**, Frequencies of tumor antigen-specific CD8 T cells (*n* *=* 23 epitopes from nine patients) in conventional (*x* axis) and NeoScreen (*y* axis) cultures. **g**, Frequencies of antigen-specific CD8 T cells (*n* *=* 23) in in vitro expanded TIL cultures (2×: re-stimulated). Box plots represent the median (line), 25% and 75% confidence limit (box limits) and min to max (whiskers). In **c**,**f** and **g**, the background levels of 4-1BB expressed by cognate negative controls were subtracted. In **c** and **f**, the highest values between 1×NeoScreen and 2×NeoScreen are considered, and data are displayed in logarithmic scale. In **c** and **e**–**g**, *P* values were determined with one-tailed paired *t*-tests.

**Extended Data Fig. 1:**
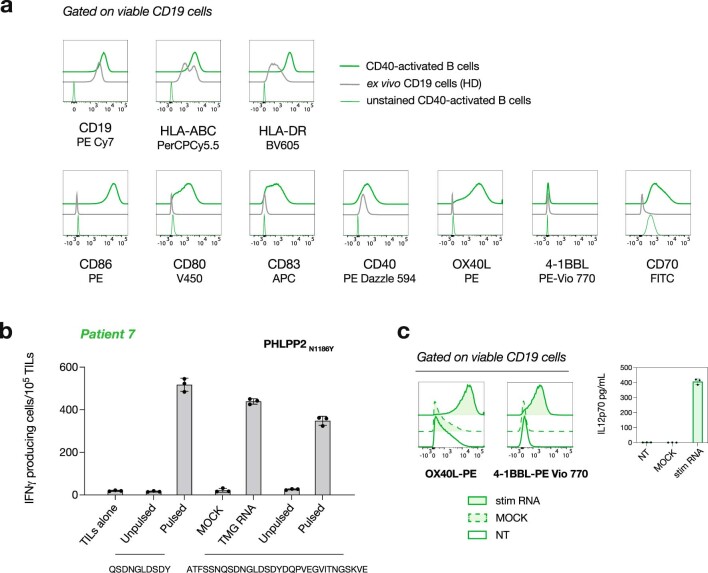
Phenotype and potency of APCs. **a**, Representative profiling of viable CD40-act B. *Ex vivo* peripheral CD19 cells from a healthy donor (HD) were used as control. **b**, Comparison of the level of neoepitope-specific T cell stimulation obtained with CD40-act B cells loaded with different sources of antigen. Autologous CD40-act B cells were either pulsed with the minimal epitope, electroporated with RNA-encoding tandem minigene (TMG) or loaded with the 31mer. B cells were co-cultured with *x1NeoScreen* TILs from patient 7 (Supplementary Table 4). T cell reactivity to PHLPP2_N1186Y_ was assessed by IFNγ ELISpot assay (*n* *=* 1 experiment, mean±SD of triplicate). MOCK: B cells transfected with PBS. **c**, CD40-act B cells electroporated with RNA encoding immune stimulatory molecules OX40L, 4-1BBL and IL-12 (Methods). Flow cytometry analysis of 4-1BBL and OX40L expression after electroporation of B cells from a representative patient (left). MSD measurement of IL-12p70 production by electroporated B cells (right, mean±SD of triplicate). MOCK: B cells transfected with PBS, NT: non-transfected, stim: stimulatory.

In this study, we developed NeoScreen, an in vitro TIL expansion and screening methodology that aims at optimizing the sensitivity of antigen validation and also isolating rare tumor antigen-specific CD8 T cells for cloning of cognate TCRs from highly enriched tumor antigen-specific CD8 T cells. Unlike conventional culture methods that rely solely on the growth factor interleukin (IL)-2, NeoScreen is based on the early exposure of TILs grown from whole tumor fragments or from dissociated tumor cells to antigens of choice^15^ loaded on competent autologous antigen-presenting cells (APCs) (Fig. 1a). We chose CD40-activated (CD40-act) B cells as APCs because they are easily procurable and expandable from low amounts of blood relative to dendritic cells and easy to engineer by electroporation. Consistently with previous studies^29^, CD40-act B cells expressed key molecules required for antigen presentation and T cell activation (Extended Data Fig. 1a). Accordingly, CD40-act B cells loaded with diverse sources of neoantigens (that is, transfected with minigenes or pulsed with synthetic peptides) ensured efficient stimulation of neoepitope-specific CD8 TILs ex vivo (Extended Data Fig. 1b). To optimize APC potency, we engineered CD40-act B cells by co-electroporation of RNA encoding immune stimulatory 4-1BB ligand (4-1BBL/CD137), OX40 ligand (OX40L/CD252) and IL-12 (ref. ^30^) (Extended Data Fig. 1c).

**Extended Data Fig. 2:**
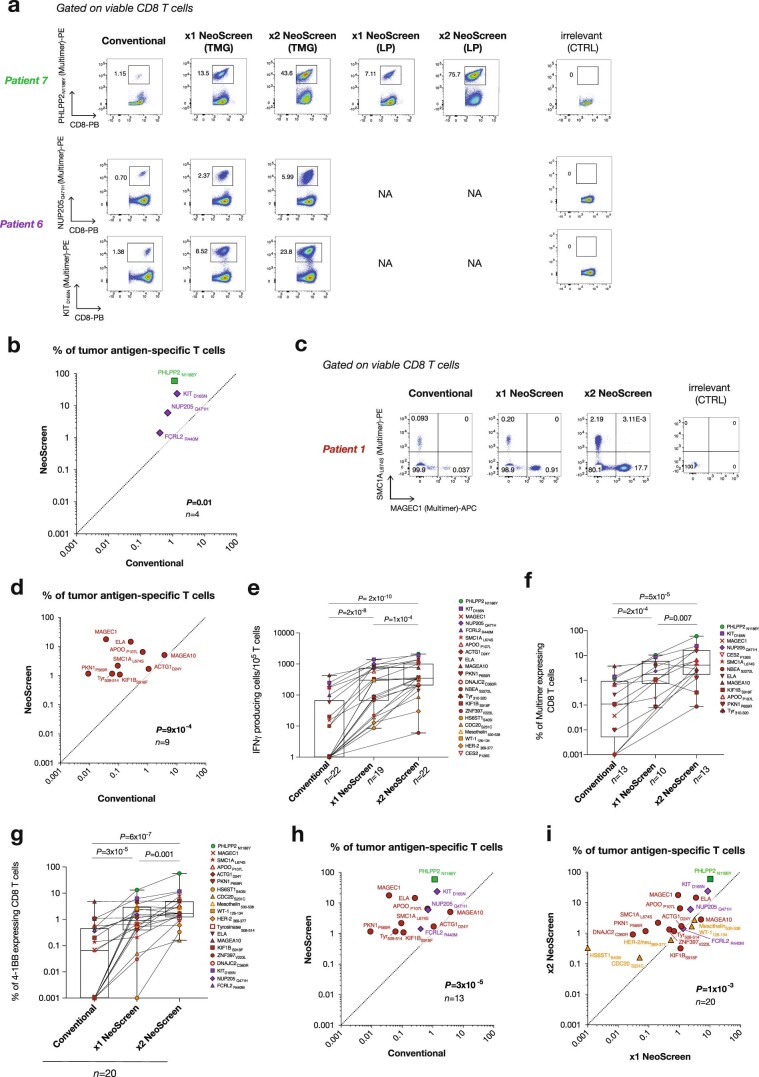
Increased detection of tumor antigen-specific CD8 T cells with *NeoScreen*. **a**, Frequency of neoepitope-specific CD8 T cells from patients 6 and 7 measured with pMHC multimers (CTRL: control, NA: not available, LP: long peptide, TMG: tandem minigene). **b**, Cumulative analysis of the frequency of tumor antigen-specific T cells (*n* *=* 4 epitopes, Supplementary Table 4) in conventional (x-axis) and *NeoScreen* (y-axis) cultures of patients 6 and 7. **c**, Representative example of the frequency of neoepitope- and TAA-specific CD8 T cells from patient 1 measured with pMHC multimers. **d**, Cumulative analysis of the frequency of tumor antigen-specific T cells (*n* *=* 9 enriched epitopes from seven patients dedicated to antigen discovery, Supplementary Table 2) in conventional (x-axis) and *NeoScreen* (y-axis) cultures, by pMHC multimers. **e-g**, Magnitude of tumor antigen-specific CD8 T cells (determined by IFNγ Spot Forming Unit per 10^5^ cells (**e**, *n* *=* 22 epitopes), pMHC-multimers staining (**f**, *n* *=* 13) or upregulation of 4-1BB (**g**, *n* *=* 20)) obtained with *NeoScreen* or conventional cultures. Box plots represent median (line), 25% and 75% confidence limit (box limits) and min to max (whiskers). **h**, Cumulative frequencies of tumor antigen-specific T cells, for enriched epitopes only (*n* *=* 13 epitopes from all nine patients) in conventional (x-axis) and *NeoScreen* (y-axis) cultures, by pMHC multimers or 4-1BB up-regulation. **i**, Cumulative frequencies of tumor antigen-specific T cells (*n* *=* 20 epitopes from all nine patients) in x1*NeoScreen* (x-axis) and x2*NeoScreen* (y-axis) cultures, by pMHC multimers or 4-1BB up-regulation. In **d**, **e**, **g-i**, the background levels of IFNγ Spot Forming Unit (**e**) or 4-1BB expression (**d**, **g-i**) by cognate negative controls (TILs alone) were subtracted. In **b**, **d**, **h** and **i**, the highest values between 1x*NeoScreen* and 2x*NeoScreen* are considered and data are displayed in logarithmic scale. In **b**, **d** and **e-i**, *P-values* were determined with one-tailed paired t-tests.

As proof of principle, we first validated the contribution of the Neoscreen approach by interrogating TILs from two tumor specimens (patients 6 and 7; Supplementary Tables 1 and 4) where we could readily identify four neoepitope reactivities among (conventional) TILs expanded with IL-2. As compared to conventional TILs, we detected markedly increased frequencies of neoepitope-specific CD8 T cells among TILs exposed to autologous engineered APCs (*P* *=* 0.01, *n* *=* 4; Extended Data Fig. 2a,b).

We then tested the ability of NeoScreen to reveal novel tumor antigens in seven additional patients (Supplementary Tables 1–4). We applied the proteogenomics NeoDisc pipeline (Methods) for prediction, immunopeptidomics-based identification and prioritization of neoantigens, focusing exclusively on non-synonymous somatic point mutations and tumor-associated antigen (TAA) candidates. Engineered autologous APCs loaded with neoantigens and/or TAAs candidates were added once (1×) or twice (2×) during TIL stimulation, and NeoScreen-expanded TILs were compared to conventional TIL cultures for the presence of antigen-specific cells (Fig. 1a). NeoScreen enabled the identification of 19 tumor epitopes in the seven patients (Fig. 1b–e). For 9of the 19 epitopes, a significantly higher frequency of specific TILs was observed in NeoScreen relative to conventional cultures (*P* *=* 9 × 10^−4^, *n* *=* 9; Extended Data Fig. 2c,d and Fig. 1b–d), whereas, for 10 of the 19 epitopes, tumor antigen-specific TILs were exclusively found in NeoScreen TILs (Fig. 1b–d). Taken together, the average number of tumor epitopes per patient was three with NeoScreen compared to one using the conventional strategy (*P* = 0.02; Fig. 1e).

**Extended Data Fig. 3:**
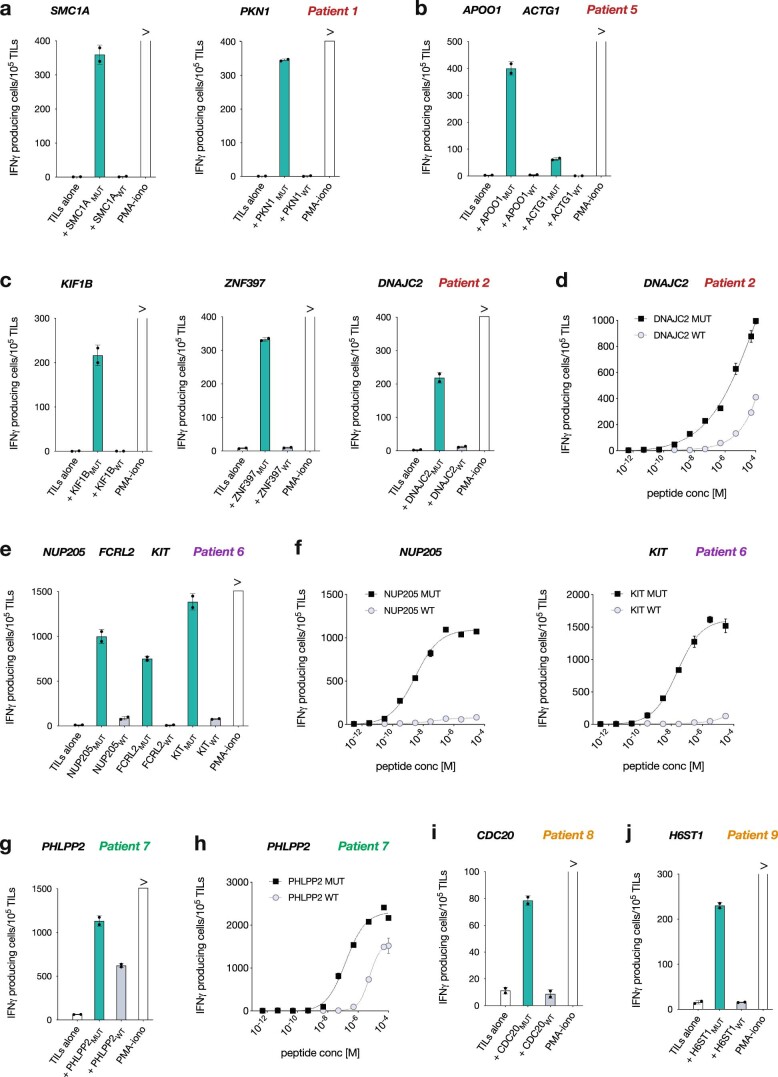
Limited cross-reactivity of neoepitope-specific CD8 T cell responses. **a-j**, Representative examples of T cell responses of *NeoScreen* TILs against mutated (MUT) vs wild type (WT) peptides (each at 1 µg/mL) by IFNγ ELISpot assay (mean±SD of duplicate). Sequences are detailed in Supplementary Table 4. Dose titration curves of T cell responses against neoepitopes and cognate WT peptide (**d**, **f** and **h**). (PMA-iono: phorbol 12-myristate 13-acetate ionomycin).

**Extended Data Fig. 4:**
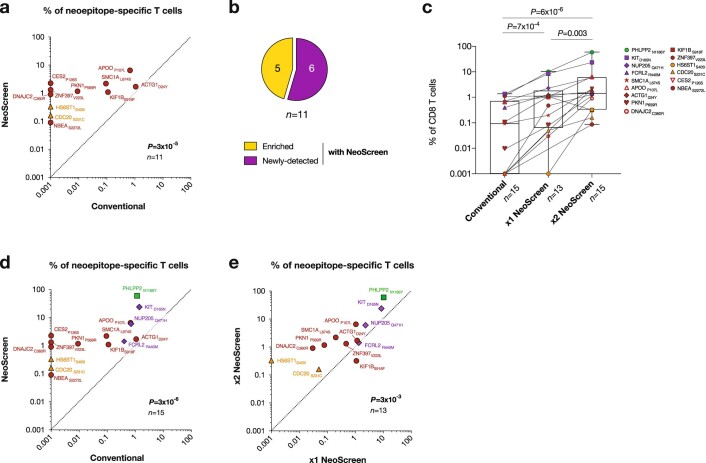
Improved identification of neoantigen-specific CD8 T cells with *NeoScreen*. **a-b**, Neoantigen discovery with *NeoScreen* (*n* *=* 6 patients, Supplementary Table 2). **a**, Cumulative analysis of the frequency of neoepitope-specific T cells (*n* *=* 11 neoepitopes, Supplementary Table 4) in conventional (x-axis) and *NeoScreen* (y-axis) cultures of patients 1, 2, 4, 5, 8 and 9, by pMHC multimers staining or 4-1BB up-regulation. **b**, Proportion of neoepitopes among enriched *versus* newly-detected T cell reactivities. **c**, Cumulative frequencies of neoepitope-specific CD8 T cells (*n* *=* 15 neoepitopes from 8 patients) in *in vitro*-expanded TIL cultures (x2: re-stimulated). Box plots represent median (line), 25% and 75% confidence limit (box limits) and min to max (whiskers). **d**, Frequencies of neoepitope-specific CD8 T cells (*n* *=* 15) in conventional (x-axis) and *NeoScreen* (y-axis) cultures. **e**, Cumulative frequencies of neoepitope-specific T cells (*n* *=* 13 neoepitopes) in x1*NeoScreen* (x-axis) and x2*NeoScreen* (y-axis) cultures. In **a**, **c-e**, the background levels of 4-1BB expression by cognate negative controls were subtracted. In **a** and **d**, the highest values between 1x*NeoScreen* and 2x*NeoScreen* are considered. In **a** and **c-e**, *P-values* were determined with one-tailed paired t-tests and data are displayed in logarithmic scale.

**Extended Data Fig. 5:**
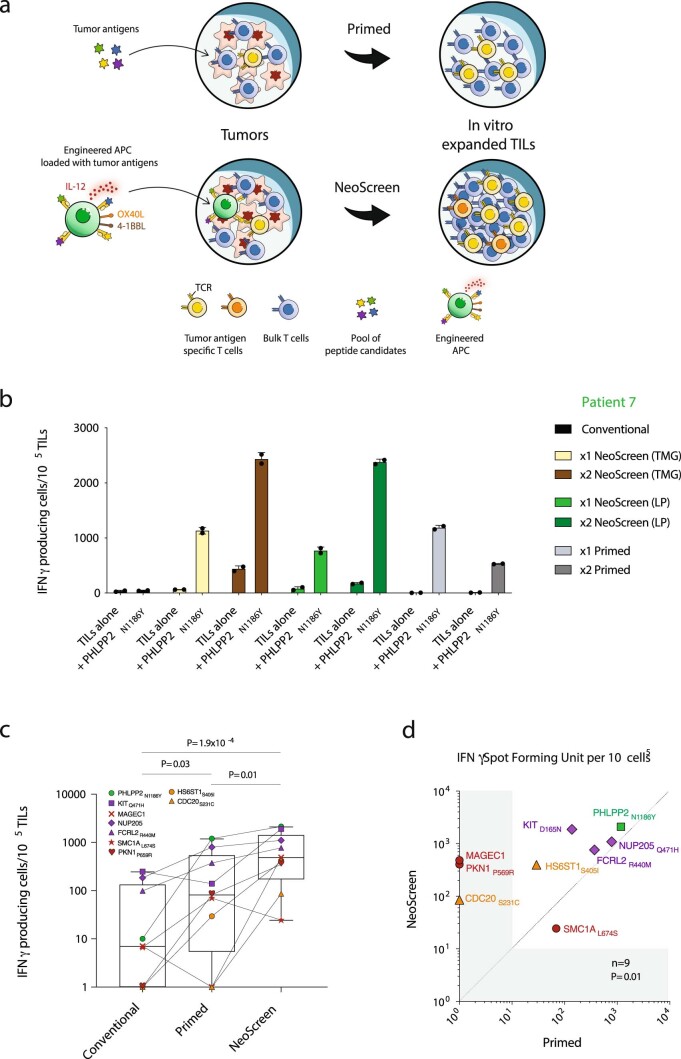
Added value of the presence of engineered B cells in *NeoScreen* - Increased sensitivity of *NeoScreen* over peptides alone (*Primed*^15^*)* for antigen discovery. **a**, Comparison of *NeoScreen* to *Primed*, based on the addition of peptide pools (in the absence of APC) at the initiation of TIL cultures. **b**, Potency of re-stimulation of TILs by *Primed* versus *NeoScreen* for patient 7. Frequency of neoantigen-specific T cells was determined by IFNγ Spot Forming Unit per 10^5^ cells (mean±SD of duplicate) following re-challenge with PHLPP2_N1186Y_ peptide. **c**, Magnitude of tumor antigen-specific T cells determined by IFNγ Spot Forming Unit per 10^5^ cells (*n* *=* 9 epitopes) obtained with *NeoScreen*, *Primed* or conventional cultures. Box plots represent median (line), 25% and 75% confidence limit (box limits) and min to max (whiskers). **d**, Cumulative analysis of the frequency of antigen-specific T cells (*n* *=* 9 tumor epitopes, Supplementary Table 4) in *Primed* (x-axis) and *NeoScreen* (y-axis) cultures of patients 1, 6, 7, 8 and 9 (Supplementary Table 2), by IFNγ Spot Forming Unit per 10^5^ cells. For **c** and **d**, the background levels of IFNγ Spot Forming Unit by cognate negative controls (TILs alone) were subtracted and the highest values between 1x*NeoScreen* and 2x*NeoScreen* are considered. *P-value*s were determined with one-tailed paired t-tests and data are displayed in logarithmic scale.

Cumulatively, through NeoScreen, using IFNγ enzyme-linked immunospot (ELISpot), pMHC-multimer and 4-1BB staining, we validated a total of 23 tumor antigens (Supplementary Table 4), including 15 neoepitopes (Extended Data Fig. 2e–g). Consistently with previous studies^15,17,18^, neoantigen-specific TILs exhibited no or limited cross-reactivity against cognate wild-type peptides (Extended Data Fig. 3). Relative to conventional TIL cultures, NeoScreen TILs were significantly enriched by several orders of magnitude for cells reactive to neoepitopes or TAAs (*P* = 7 × 10^−9^, *n* *=* 23; Fig. 1f). The frequency of TILs targeting epitopes identified in both NeoScreen and conventional conditions was increased by ~67-fold (*P* = 3 × 10^−5^, *n* *=* 13 epitopes; Extended Data Fig. 2h). Of interest, a second round of TIL stimulation further increased their frequency (Fig. 1g and Extended Data Fig. 2i). Of note, NeoScreen remains significantly superior to the conventional strategy when exclusively neoantigens are considered (Extended Data Fig. 4). Also, NeoScreen was found to be significantly improved relative to our previous study^15^ using peptides alone (Extended Data Fig. 5). Overall, engineered APCs in the presence of tumor antigens enabled the substantial expansion of neoantigen (and TAA)-specific CD8 T cells in melanoma and in ovarian, lung and colon cancer, thus establishing a highly sensitive and reproducible methodology to identifying tumor antigens.

**Fig. 2:**
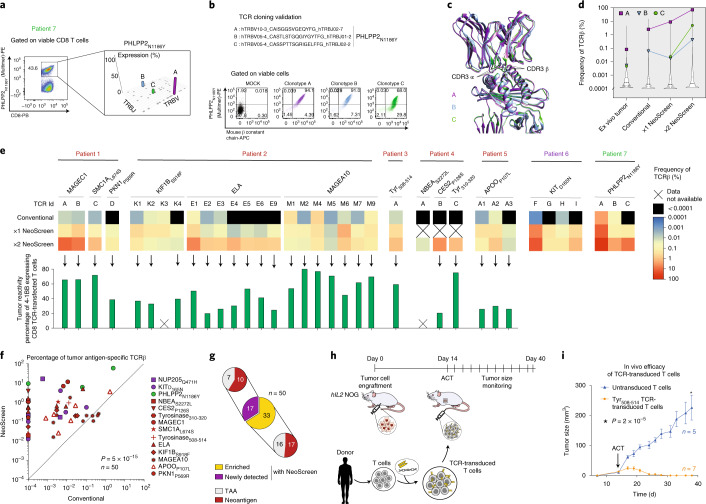
Tumor-reactive TCR identification and validation. **a**, Representative example of neoepitope-specific CD8 T cell sorting by pMHC-multimer. Manhattan plot depicts TCRβ chain VJ recombination of PHLPP2_N1186Y_-specific clonotypes A, B and C. **b**, Validation of antigen specificity after TCR cloning. **c**, Superimposition of the modeled TCR-pMHC complexes for TCR-A, TCR-B and TCR-C. The location of CDR3α and CDR3β loops is shown by arrows. **d**, Violin plots display frequencies of TCRβ-A, TCRβ-B and TCRβ-C in bulk TCR repertoires of the different TIL cultures and of the original tumor. **e**, Heat maps depict the frequencies of tumor antigen-specific TCRβ clonotypes (*n* *=* 50) within the different bulk TIL populations (top). Overview (bottom) of tumor reactivity of TCR-transfected primary CD8 T cells (*n* *=* 31 and Extended Data Fig. 10a). The background levels of 4-1BB expressed by cognate negative controls (TCR T cells alone) were subtracted (Supplementary Fig. 4). In **d** and **e**, NeoScreen TILs from patient 7 were generated with long peptides. **f**, Cumulative analysis of the frequency of tumor antigen-specific TCRβ detected in conventional (*x* axis) and NeoScreen (*y* axis) cultures. Highest values between 1×NeoScreen and 2×NeoScreen are considered, and data are displayed in logarithmic scale. *P* value was determined with a one-tailed paired *t*-test. **g**, Proportions of neoepitope- versus TAA-specific TCRβ among enriched versus newly detected clonotypes. **h**, ACT of TCR-transduced T cells in autologous patient-derived xenograft tumor model. **i**, In vivo efficacy of adoptively transferred tyrosinase_508–514_ TCR-transduced T cells against autologous patient-derived tumor xenografts. The graph shows tumor size (mean ± s.e.m. of replicates) over time. *P* value was determined with a one-tailed unpaired *t*-test.

**Extended Data Fig. 6:**
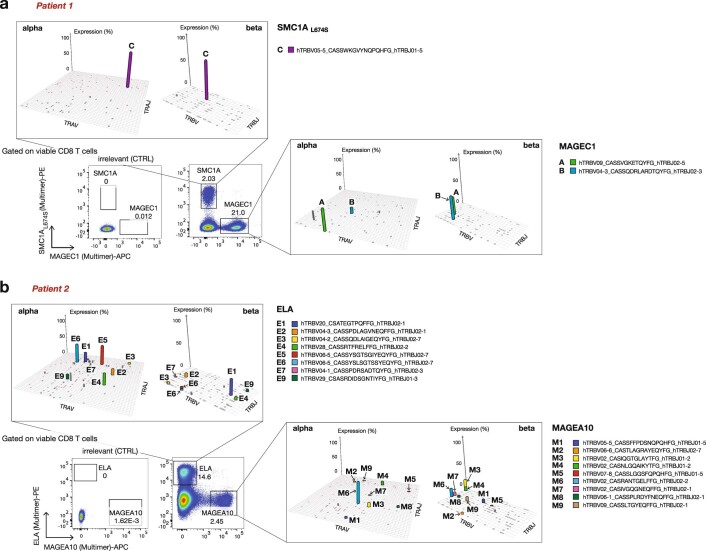
Identification of tumor antigen-specific TCRs. Representative examples of TCR repertoire analyses upon isolation of antigen-specific T cells by FACS. Tumor antigen-specific T cells from patient 1 and 2 x2*NeoScreen-*stimulated TILs were FACS sorted using pMHC multimers and immediately processed for TCR bulk sequencing analysis (TCRα and TCRβ chains). Tumor antigens and cognate TCRs are described in Supplementary Tables 4 and 5. Manhattan plots report TCRα and TCRβ V/J recombinations of tumor antigen-specific T cells: V and J segments are represented according to chromosomal location on x and y-axes, respectively.

**Extended Data Fig. 7:**
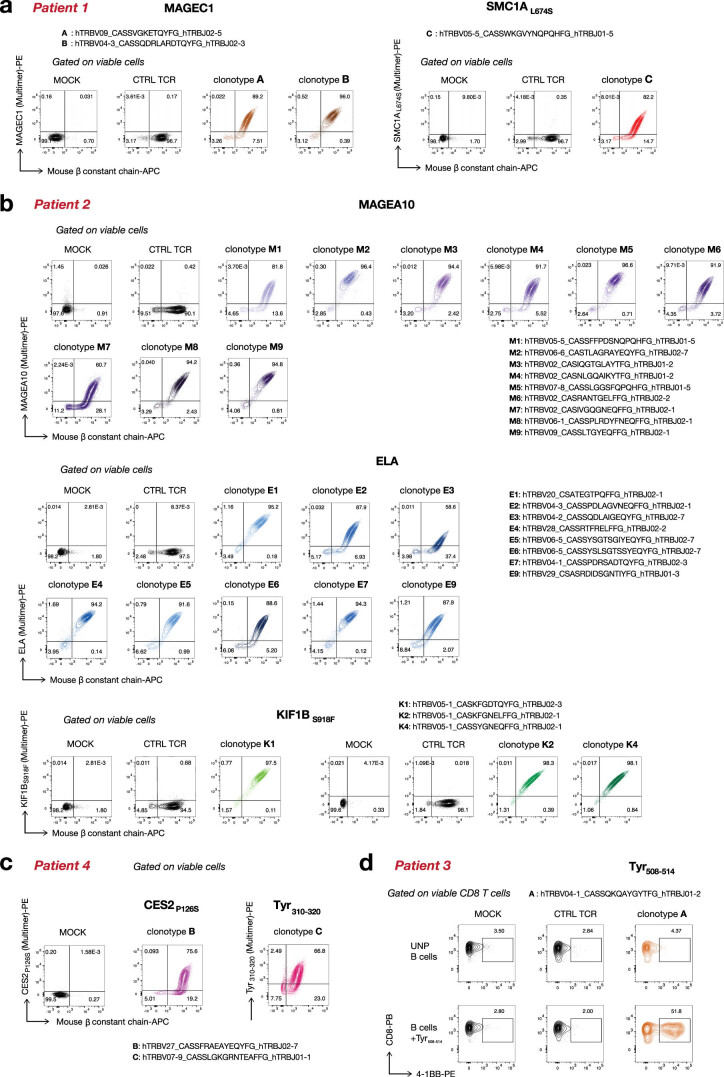
Validation of antigen-specific TCRs upon TCR cloning. **a-c**, Representative examples of validation of TAA- and neoepitope-specific TCRαβ pairs from patients 1, 2 and 4. **a-c**, Validation of tumor antigen-specificity after labeling with cognate pMHC multimers of Jurkat cells co-electroporated with TCRα and TCRβ chain RNAs. Dot plots report the concomitant expression of the transgenic TCR and of the mouse TCRβ constant region used as a marker of transfection efficiency. **d**, For patient 3, tyrosinase_508-514_-specific TILs were FACS-sorted based on 4-1BB upregulation. Autologous activated primary T cells cloned with TCRαβ pair were co-cultured with autologous CD40-act B cells pulsed with peptide LPEEKQPL. Reactivity was assessed by 4-1BB upregulation. MOCK: control of transfection, neg pair: irrelevant TCRα/β pair, UNP: unpulsed, no antigen.

**Extended Data Fig. 8:**
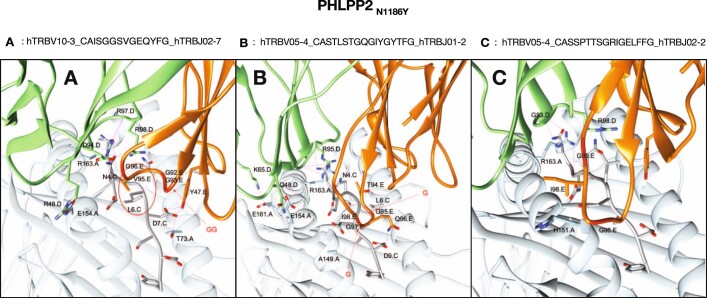
Molecular modelling of PHLPP2_N1186Y_-specific pMHC-TCRs interactions. Modelled structures of the three PHLPP2_N1186Y_- specific TCRs (TCR-A, -B, and -C, Supplementary Table 6) showing detailed predicted interactions with a cognate pMHC complex. TCRα ribbon is colored in light green, with relevant interacting residues displayed in sticks. Atoms are colored according to the atom types, with the exception of carbon atoms that are colored in light green. TCRβ is colored in orange, with relevant interacting residues displayed in sticks and atoms colored according to the atom types, with carbon colored in orange. MHC (HLA-A*01:01) is colored in light blue, with residues displayed in sticks and atoms colored according to the atom types, with carbon colored in light blue. The peptide is shown in grey sticks and atoms colored according to the atom types and carbon colored in grey. Residues are labelled in black.

We next theorized that this novel platform would enable sensitive isolation of relevant TCRs directed against private tumor antigens (Fig. 1a). We purified tumor antigen-specific NeoScreen TILs using pMHC-multimers or 4-1BB upregulation and performed bulk TCRα and TCRβ sequencing of isolated T cells (Fig. 2a, Extended Data Fig. 6 and Supplementary Table 5). Individual tumor epitopes were recognized by one or more clonotypes, occurring at different frequencies among NeoScreen TILs. To confirm the specific recognition of tumor antigens, TCRαβ pairs were cloned into recipient Jurkat cells or primary T cells, which were then interrogated for expression of functional TCRs by pMHC-multimers (Fig. 2b and Extended Data Fig. 7a–c) or 4-1BB upregulation (Extended Data Fig. 7d). Figure 2b shows an example of functional validation of three distinct TCRs (A, B and C) cloned from sorted PHLPP2_N1186Y_-specific NeoScreen TILs. In addition, analysis of the three-dimensional TCR-pMHC structures obtained by homology modeling indicates that all three PHLPP2_N1186Y_-specific TCRs could establish interactions with the cognate pMHC (Fig. 2c, Extended Data Fig. 8 and Supplementary Table 6).

**Extended Data Fig. 9:**
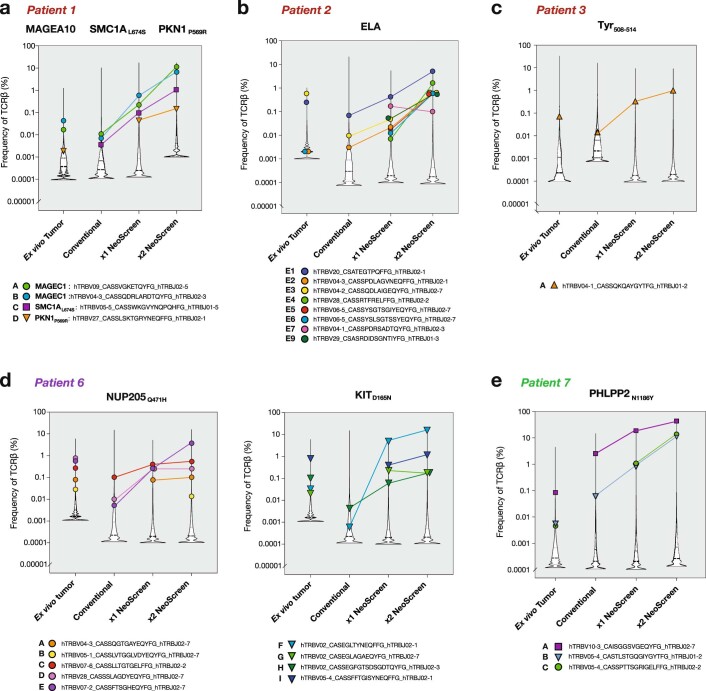
Tracking of antigen-specific TCRs in *ex vivo* and *in vitro*-expanded TIL samples. **a-e**, TCRβ repertoire analysis was performed on *ex vivo* tumors, bulk conventional TILs and *NeoScreen* expanded TILs. The frequency of each tumor antigen-specific TCRβ within the bulk TIL populations and within *ex vivo* tumors, validated as shown in Extended Data Figure 6, is evidenced by colored symbols. Representative examples of TCR tracking from patients 1-3, 6 and 7 are displayed. For patient 7, *NeoScreen* TILs generated with tandem minigene are shown (Methods). Violin plots report, in each sample, the bulk TCR repertoire distribution, as well as the frequency of tumor antigen-specific TCRβ clonotypes. (x2: re-stimulated).

**Extended Data Fig. 10:**
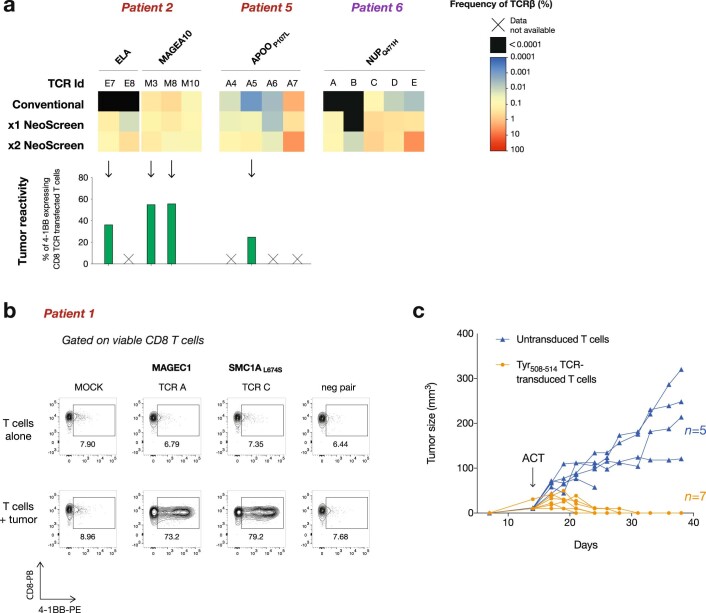
Frequency, reactivity and efficacy of tumor-reactive TCRs. **a**, Heatmaps reporting the frequencies of antigen-specific TCRβ clonotypes from patients 2, 5 and 6 within the different bulk TIL populations (top). TCRs are detailed in Supplementary Table 5. Antitumor-reactivity of TCR-transfected primary CD8 T cells, measured by 4-1BB upregulation following co-culture with autologous tumor cells (bottom). The background levels of 4-1BB expressed by cognate negative controls (TCR-T cells alone) were subtracted (Supplementary Fig. 4). **b**, Representative example of flow cytometry data showing *in vitro* tumor recognition (4-1BB upregulation) of antigen-specific TCRs (MAGEC1 TCRs A and B and SCM1A_L674S_ TCR C) from patient 1. (MOCK: control of transfection, neg pair: irrelevant TCRα/β pair). **c**, *In vivo* efficacy of adoptively-transferred tyrosinase_508-514_ TCR-transduced T cells against autologous patient-derived tumor xenografts. The graph shows tumor size of individual *hIL-2* NOG mice adoptively-transferred with TCR-transduced (in orange; *n* *=* 7) and untransduced (in blue; *n* *=* 5) cells. ACT was performed on Day 14.

We next performed TCRβ sequencing of bulk TIL cultures and ex vivo tumors (when available). We ascertained that the NeoScreen process indeed led to marked expansion of tumor antigen-specific TILs through tracking validated TCRβ sequences in the original tumor and in NeoScreen-expanded TILs (Fig. 2d,e and Extended Data Fig. 9). As shown for representative PHLPP2_N1186Y_-specific TCRs, all three TCRs were detected in the original tumor, and their respective frequencies considerably increased in NeoScreen-expanded TILs (Fig. 2d,e and Extended Data Fig. 9e). Of interest, although TCR-B and TCR-C were detected at similar frequencies (~0.005%) in the original tumor, only TCR-B was found in conventional TILs, TCR-C being likely diluted under conventional culture conditions or only mobilized under NeoScreen conditions (Fig. 2d). Cumulative data of 50 clonotypes confirmed the potential of NeoScreen to identify novel TCRs specific to neoantigens or TAAs that were not detected in conventional TILs (*n* *=* 17/50; Fig. 2e–g and Extended Data Figs. 9 and 10a). Overall, we demonstrated a considerable enrichment of tumor antigen-specific TCRs by several orders of magnitude in NeoScreen TILs over conventional TILs (*P* = 5 × 10^−15^, *n* *=* 50; Fig. 2f and Extended Data Figs. 9 and 10a).

Although neoantigen-specific TILs have been associated with clinical responses to immune checkpoint blockade^31^ and TIL ACT^3,32^, the recognition of autologous tumors by neoantigen-specific TCRs^13,26,33^ has not been consistently investigated. We, thus, interrogated the anti-tumor reactivity of validated tumor antigen-specific TCRs revealed by NeoScreen, when autologous tumor cell lines were available (Extended Data Fig. 10b). Upon TCR cloning in primary activated T cells, all NeoScreen-derived TCRs (*n* *=* 31) specific to neoepitopes or TAAs were found to be tumor reactive (Fig. 2e, Extended Data Fig. 10a and Supplementary Fig. 4). To our knowledge, this is the first extensive demonstration that neoantigen-specific TCRs consistently target autologous tumors.

Finally, we tested the hypothesis that TCRs identified with NeoScreen could be used for individualized TCR-based ACT. Using patient-derived xenograft tumors in the human IL-2 transgenic (*hIL-2*) NOG mouse model^34^, we showed that adoptively transferred peripheral blood T cells transduced with tumor antigen-specific TCR cloned from NeoScreen TILs mediated specific regression of established tumors in vivo (Fig. 2h,i and Extended Data Fig. 10c). Taken together, our data demonstrate in vitro and in vivo anti-tumor reactivity of antigen-specific TCRs identified through NeoScreen. This supports the feasibility of using NeoScreen for TCR gene transfer therapy.

Here we report NeoScreen, a method that enables highly sensitive screening of tumor (neo)antigens and yields a markedly broader repertoire of tumor antigen-reactive TCRs than has been possible to date. NeoScreen acts not only by increasing the frequency of antigen-specific TCRs found with conventional methods but also by recruiting additional TCR clonotypes that can be newly detected with markedly enhanced sensitivity. To our knowledge, this is the first time that engineered B cells have been used at the initiation of TIL growth to enrich the sensitivity of antigen discovery. RNA electroporation technology makes our approach easily applicable and offers the possibility to further engineer APCs for future improvements. Notably, although the requirement to generate autologous B cells and to predict and synthesize antigens delays the initiation of NeoScreen in vitro cultures by a couple of weeks, timelines remain in the same overall range as compared to alternative strategies with limited sensitivity. In this study, we focused on MHC class I restricted antigen discovery. However, the strategy could potentially be modified to also permit the identification of CD4 T cell responses, given their emerging clinical relevance^1,35^. Of note, we cannot exclude that TIL stimulation with antigen-loaded APCs might potentially lead to the recruitment of clonotypes of lower avidity than those mobilized with the conventional strategy. In addition, because the purpose of NeoScreen is to generate TIL populations enriched in selected tumor antigens, it skews the repertoire to reveal the presence of neoepitope-reactive clones in tumors. Our data suggest that this bias might shift the TIL repertoire toward enrichment in tumor-reactive, antigen-specific clonotypes, potentially representing improved TIL products for ACT. Overall, NeoScreen enables the highly efficient identification of tumor-specific antigens in melanoma, as well as in ovarian, colorectal and lung cancer, and also enables the highly sensitive isolation of cognate tumor-reactive TCRs. Thus, NeoScreen represents a valuable pipeline to select relevant private target antigens for cancer vaccines and isolate tumor-reactive TCRs for personalized engineered T cell therapy of solid tumors.

## Methods

### Patient

This study included patients with stage III/IV metastatic melanoma and patients with ovarian cancer, non-small cell lung cancer and colorectal cancer, all of whom had received several lines of chemotherapy (Supplementary Table 1). Patients were enrolled under protocols approved by the respective institutional regulatory committees at the University of Pennsylvania and Lausanne University Hospital (Ethics Committee, University Hospital of Lausanne-CHUV). Also, samples from four patients with melanoma enrolled in a phase I clinical trial of TIL ACT were collected at baseline (NCT03475134). All patients provided informed consent.

### Tumors and blood processing

Resected tumors were minced into 1–2 mm^2^ pieces or enzymatically digested and cryopreserved in 90% human serum + 10% dimethyl sulfoxide (DMSO) as described^15,36^. Both enzymatically digested tumor cells and tumor fragments were used as starting material for TIL generation. Peripheral blood mononuclear cells (PBMCs) were isolated from leukapheresis upon thawing and washing using the Lovo spinning membrane filtration system (Frenesius Kabi). PBMCs were cryopreserved in 90% human serum + 10% DMSO.

### Generation of tumor cell lines

Tumor cell lines were established from tumor fragments and cultured in R10 medium (RPMI 1640 complemented with 10% FBS, 100 mM HEPES (Gibco), 100 IU ml^−1^ of peninicillin and 100 μg ml^−1^ of streptomycin (Bio-Concept)) at 37 °C at 5% CO_2_. Culture medium was replenished every 2–3 d, and cultures were split when confluent. To this end, tumor cells were gently detached with Accutase (Thermo Fisher Scientific) and split, and R10 medium was fully replenished. The day before any co-culture assay (screening assay described below), tumor cells were incubated for 24 h in R10 medium supplemented with 200 ng ml^−1^ of IFNγ (Miltenyi Biotec).

### Generation and electroporation of APCs

B cells were isolated from autologous cryopreserved PBMCs or apheresis samples by positive selection of CD19 cells with microbeads (Miltenyi Biotec). CD19 cells were then cultured at 37 °C at 5% CO_2_ for 7 to ~20 d in R8 medium (RPMI 1640 (Gibco) with 8% human AB serum (Bio West), non-essential amino acids, 100 mM HEPES, 1 mM sodium pyruvate, 50 μM 2-mercaptoethanol (Gibco), 100 IU ml^−1^ of penicillin, 100 μg ml^−1^ of streptomycin (Bio-Concept) and 2 mM L-glutamine solution (Bio-Concept)), supplemented with 0.5–1 μg ml^−1^ of multimeric CD40L (AdipoGen), with 40 ng ml^−1^ of IL-4 (Miltenyi Biotec) and 50 ng ml^−1^ of IL-21 (Miltenyi Biotec). Between days 7 and 14, B cells were harvested and either used for screening or TIL generation or frozen for future use. For flow cytometry phenotyping analysis, day 9–12 B cells were stained with anti-human CD19, CD80, OX40L, CD70 (BD Biosciences), HLA-ABC, HLA-DR, CD40, CD83, CD86 (BioLegend), 4-1BBL (Miltenyi Biotec) (Supplementary Methods) and Aqua viability dye (Thermo Fisher Scientific) in two distinct fluorescence-activated cell sorting (FACS) panels, acquired on a four-laser Fortessa (BD Biosciences) with FACS DIVA software v.9.0 (BD Biosciences) and analyzed with FlowJo X (TreeStar).

The secretion of IL-12 by B cells was assessed by MSD immunoassay (Human Cytokine 30-Plex Kit, Meso Scale Discovery), according to the manufacturer’s instructions, and was analyzed with the MESO QuickPlex SQ 120 instrument (Meso Scale Discovery).

Before electroporation, B cells were rested overnight in their culture medium including cytokines, without CD40L. Cells were electroporated using both the Neon transfection 10 μl and 100 μl kits (Thermo Fisher Scientific). Briefly, B cells were harvested, washed twice and resuspended at 10–20 × 10^6^ cells per ml in buffer T. B cells were mixed with 100 μg ml^−1^ of in vitro transcription (IVT) tandem minigene (TMG) RNA and/or with 33 μg ml^−1^ of each immune stimulatory IVT RNA. Cells were then electroporated in 10-μl (0.1–0.2 × 10^6^ cells) or 100-μl (1–2 × 10^6^ cells) tips with the following parameters: 1,400 V, 10 ms, three pulses. After transfection, cells were added to pre-warmed medium and either incubated for 2–17 h (overnight) at 37 °C or used immediately.

### Identification of non-synonymous tumor mutations and prediction of neoantigens

Non-synonymous point tumor mutations arising from single-nucleotide variants were identified from tumor tissues and matched healthy tissues. Samples from patients 4, 6, 7, 8 and 9 were analyzed as previously described^15^. Samples from patients 1, 2, 3 and 5 were analyzed with NeoDisc v.1.2 pipeline^37^ that includes the GATK^38^ variant calling algorithms Mutect2, Mutect1, HaplotypeCaller and VarScan 2. NeoDisc v.1.2 also determines the presence of each mutation and quantifies the expression of each mutant gene and mutation from RNA sequencing data. Predictions for binding to HLA class-I of all candidate peptides of samples from patients 4, 6, 7, 8 and 9 were performed using the netMHC v.3.4 and netMHCpan-3.0 (refs. ^39,40^) algorithms. Predictions for binding and immunogenicity on HLA class-I and HLA-class II candidate peptides of samples from patients 1, 2, 3 and 5 were performed using the PRIME^41^ and MixMHCpred2 algorithms^42,43^. Long peptides consisted of 31mers with the mutation at the center position for samples from patients 4 and 7, and peptides were optimally designed, as described, for samples from patients 1, 2, 3 and 5 (ref. ^37^). Long and short peptides analyzed with NeoDisc v.1.2 were selected based on their binding and immunogenicity predictions, the expression of the mutant genes, the expression of the mutations and the presentation of the peptides in IpMSDB (a database of hotspots of antigen presentation)^44^.

For HLA typing, genomic DNA was extracted from samples using the DNeasy kit from Qiagen. HLA typing was performed with the TruSight HLA v.2 Sequencing Panel from CareDx. Briefly, 400 ng of genomic DNA was used to amplify HLA genes by polymerase chain reaction (PCR). Nextera adapters were added by tagmentation, and the resulting libraries were sequenced on the MiniSeq instrument (Illumina). Sequencing data were then analyzed with the Assign TruSight HLA v.2.1 software provided by CareDx.

### Identification of TAAs by immunopeptidomics

Immunoaffinity purification of HLA-I complexes from tissues was performed as previously described^45^ with the anti-HLA-I W6/32 antibody. HLA-I-binding peptides were eluted with 1% TFA and concentrated. Peptides were measured with a liquid chromatography with tandem mass spectrometry (LC–MS/MS) system consisting of an Easy-nLC 1200 and the Q Exactive HF-X mass spectrometer (Thermo Fisher Scientific). With the MaxQuant computational environment^46^, we searched the immunopeptidomics MS data against the patient-specific customized reference database as previously described^47^. The enzyme specificity was set as unspecific, and peptides with a length between 8 and 25 amino acids were allowed. A false discovery rate (FDR) of 5% was required for peptides, and no protein FDR was set. Peptides derived from known TAAs were selected for further analysis.

### Design of DNA constructs and in vitro transcription of RNA

TMGs were in silico designed as previously described^2,48,49^ and codon optimized and synthesized by gene synthesis at GeneArt (Thermo Fisher Scientific). Briefly, five minigenes by 31mer each were centered on identified mutated amino acids and spaced by non-immunogenic glycine/serine linkers^2,48^. Resulting TMGs were flanked by a signaling peptide and by MHC-class I trafficking signals^49^.

To get OX40L-, 4-1BBL- and IL-12 (α/β)-expressing vectors, full-length sequences coding for each immune stimulatory molecule were cloned into pcDNA™6/*myc*-His-C for OX40L and 4-1BBL (Thermo Fisher Scientific) and pGEM-T (Promega) for IL-12, downstream of a T7 promoter. Plasmids encoding OX40L, 4-1BBL and IL-12 were linearized respectively with Eco RV., Sma I. (New England Biolabs) and Xba I (Thermo Fisher Scientific).

For the TCR cloning methodology, DNA sequences coding for full-length TCR chains were codon optimized and synthesized by GeneArt (Thermo Fisher Scientific) as strings. Each DNA sequence included a T7 promoter upstream of the ATG codon, whereas human constant regions of α and β chains were replaced by corresponding homologous murine constant regions.

Linearized plasmidic DNA and purified PCR products served as templates for the IVT and polyadenylation of RNA molecules as per the manufacturer’s instructions (Thermo Fisher Scientific). Polyadenylation and integrity were assessed by gel electrophoresis in denaturing conditions, and RNA was quantified with a Qbit fluorometer (Thermo Fisher Scientific). Purified RNA was resuspended in water at 1–10 μg ml^−1^ and stored at −80 °C until used.

### Peptide loading

Peptides (purity >70%) were synthetized and lyophilized by the Peptide and Tetramer Core Facility of the Department of Oncology at UNIL-CHUV (Lausanne, Switzerland) or by Covalab (Lyon, France).

For minimal epitope loading (that is, 9–10mer), cells were harvested, washed twice with RPMI medium and resuspended at 1 × 10^6^ cells per ml in RPMI complemented with 1% human serum and with individual peptides or peptide pools at 1 μg ml^−1^. APCs were incubated at 37 °C for 1–2 h and washed twice with RPMI medium before use in co-culture assays.

For long peptide (that is, 31mer) pulsing, APCs were harvested, washed twice with RPMI medium and resuspended at 1 × 10^6^ cells per ml in R8 medium complemented only with cytokines. Peptides were added at 1 μg ml^−1^. APCs were then incubated at 37 °C for 17–20 h and washed twice with RPMI medium before use in co-culture assays.

### TIL cultures

Conventional TILs were grown in R8 medium supplemented with 6,000 IU ml^−1^ of IL-2 (Proleukin). Next, 2–6 tumor fragments (1–3 mm^3^) or a total of 1 × 10^6^ dissociated tumor cells were plated per well of a p24-well plate. In addition to tumor samples and high dose of IL-2, NeoScreen TILs were generated by the addition of engineered B cells presenting tumor antigen candidates at day 0 of culture. Antigens were in the form of minigenes or pools of predicted peptides (≤139) at 1 μg ml^−1^ each. For patient 4, a total of 191 peptides were split into two pools, noted as follows: NeoScreen (1) and NeoScreen (2) (Supplementary Tables 2 and 4). Then, 1 × 10^6^ and 2 × 10^6^ B cells were added per well of the p24-well plate with dissociated tumor cells and tumor fragments, respectively. Cells were cultured at 37 °C at 5% CO_2_ and maintained at a concentration of 1 × 10^6^ cells per ml. At days 7–10, TILs were harvested, counted and washed, and a fraction of NeoScreen TILs underwent a second round of stimulation with B cells (that is, a stimulation setting identical to day 0). After 16–22 d, TILs were collected, screened, TCR sequenced and cryopreserved.

### Antigen screening of TIL cultures

IFNγ ELISpot and pMHC-multimer complexes staining were performed at the end of cultures, and antigens were validated by three or more independent experiments. For patient 4, NeoScreen (1) and NeoScreen (2) were interrogated each with corresponding antigen candidates, added at the initiation of TIL generation. For patient 7, NeoScreen TILs were generated (1×) and re-stimulated (2×) in parallel with TMGs and long peptides-loaded, engineered CD40-act B cells so the frequency of antigen-specific TILs obtained was averaged between the two antigen sources, unless specified.

ELISpot assays were performed using pre-coated 96-well ELISpot plates (Mabtech), as previously described^15^. Briefly, 5 × 10^4^ to 2 × 10^5^ TILs were plated per well and challenged with tumor-specific peptides at 1 µg ml^−1^ (single peptides or peptide pools of ≤139 peptides) (see example in Supplementary Fig. 1a). The background level of IFNγ spot-forming units per 10^5^ cells by the negative control (TILs alone) was subtracted from that of antigen-re-challenged TILs in all cumulative figures. The cross-reactivity of neoepitope-specific T cell responses was assessed by challenging TILs with the wild-type peptide at 1 µg ml^−1^. Cross-reactivity was then further evaluated by performing limiting peptide dilutions (ranging from 100 µg ml^−1^ to 0.1 pg ml^−1^) (Extended Data Fig. 3). When autologous B cells were used in ELISpot assay, a ratio of 2:1 TILs:APCs was applied (Extended Data Fig. 1b). Before the assay, TILs were rested for 48 h in culture medium from which IL-2 was removed in two steps. Phorbol 12-myristate 13-acetate ionomycin (Thermo Fisher Scientific) was used to stimulate TILs as positive control, and 1 × 10^3^ TILs were plated per ELISpot well.

After 16–20 h, cells were gently harvested from ELISpot plates to assess 4-1BB upregulation, and plates were developed according to the manufacturer’s instructions and counted with a Bioreader 6000-E (BioSys). Positive conditions were defined as those with an average number of spots higher than the counts of the negative control (TILs alone) plus three times the standard deviation of the negative. Cells retrieved from plates were centrifuged and stained with anti-human CD3, CD4 (BioLegend), CD8 (BD Biosciences), 4-1BB (Miltenyi Biotec) and Aqua viability dye (Thermo Fisher Scientific) (see example in Supplementary Fig. 1b and the gating strategy in Supplementary Fig. 2a and Supplementary Methods). The background levels of 4-1BB expression by the negative controls (TILs alone) were subtracted to that of antigen-re-challenged TILs in all cumulative figures.

For pMHC-multimer staining, TILs were labeled with cognate in-house pMHC-multimers (produced by the Peptide and Tetramer Core Facility of the Department of Oncology, UNIL-CHUV, Lausanne, Switzerland) and anti-CD3, -CD4 (BioLegend), -CD8 (BD Biosciences) and Aqua viability dye (Thermo Fisher Scientific) (see the gating strategy in Supplementary Fig. 2b and Supplementary Methods).

### Isolation of tumor antigen-specific T cells

Antigen-specific CD8 TILs were FACS sorted either using pMHC-multimers or based on 4-1BB upregulation^50^. For pMHC-multimer sorting, cells were stained with the Aqua viability marker (Thermo Fisher Scientific) and anti-CD4 (BioLegend) and anti-CD8 (BD Biosciences) (Supplementary Methods). For activation marker sorting, anti-human 4-1BB (Miltenyi Biotec) was used instead of the multimer (Supplementary Methods). Cell sorting experiments were performed using either a BD FACSAria II or a BD FACS Melody (BD Biosciences). Purified cells were used for TCR sequencing (see below).

Plots reporting cumulative frequencies of antigen-specific CD8 T cells in the different TIL cultures are based on pMHC-multimer data (when available; Supplementary Table 4) or 4-1BB upregulation.

### TCR α and β sequencing and analysis

mRNA was isolated using the Dynabeads mRNA DIRECT Purification Kit (Life Technologies) and was amplified using the MessageAmp II aRNA Amplification Kit (Ambion) with the following modifications: IVT was performed at 37 °C for 16 h. First, strand cDNA was synthesized using SuperScript III (Thermo Fisher Scientific) and a collection of *TRAV*/*TRBV*-specific primers. TCRs were then amplified by PCR (20 cycles with the Phusion from New England Biolabs) with a single primer pair binding to the constant region and the adapter linked to the *TRAV*/*TRBV* primers added during the reverse transcription. A second round of PCR cycle (25 cycles with the Phusion from New England Biolabs) was performed to add the Illumina adapters containing the different indexes. The TCR products were purified with AMPure XP beads (Beckman Coulter), quantified and loaded on the MiniSeq instrument (Illumina) for deep sequencing of the TCRα/TCRβ chain. The TCR sequences were further processed using ad hoc Perl scripts to (1) pool all TCR sequences coding for the same protein sequence; (2) filter out all out-frame sequences; and (3) determine the abundance of each distinct TCR sequence. TCR sequences with a single read were not considered for analysis.

### Single-cell TCR sequencing

The tumor samples were thawed on the day of the assay, and fragments were dissociated in RPMI complemented with 2% gelatin (Sigma-Aldrich), 200 IU ml^−1^ of collagenase I (Thermo Fisher Scientific), 400 IU ml^−1^ of collagenase IV (Thermo Fisher Scientific), 5 IU ml^−1^ of deoxyribonuclease I (Sigma-Aldrich) and 0.1% RNasin Plus RNase Inhibitor (Promega) for 30 min at 37 °C. Digested cells were then filtered and resuspended in PBS + 1% gelatin + 0.1% RNasin. Cells were stained first with 50 mM ml^−1^ calcein AM (Thermo Fisher Scientific) and Fc receptor blocked (Miltenyi Biotec) for 15 min at room temperature and next with anti-CD45 (BioLegend) (Supplementary Methods). Dissociated cells were resuspended in PBS complemented with 0.04% BSA + 0.1% RNasin, and DAPI (Invitrogen) staining was performed. CD45 live cells were sorted with a FACS Astrios (Beckman Coulter). Sorted cells were then resuspended at 0.6–1.2 × 10^4^ cells per μl with a viability of >90% and subjected to a 10x Chromium instrument for the single-cell analysis (10x Genomics). Next, 1.7 × 10^4^ cells were loaded per sample, with the targeted cell recovery of 1 × 10^4^ cells. Using a microfluidic technology, single cells were captured and lysed, and mRNA was reverse transcribed to barcoded cDNA (10x Genomics). Fourteen PCR cycles were performed for cDNA amplification, and a targeted enrichment for TCRs was done. VDJ libraries were obtained following the manufacturer’s instructions (10x Genomics). Barcoded VDJ libraries were then pooled and sequenced by a HiSeq 2500 sequencer (Illumina). Single-cell TCR sequencing data were processed by the Cell Ranger software pipeline (v.3.1.0, 10x Genomics).

### TCR validation

To validate antigen specificity and interrogate anti-tumor reactivity, TCRαβ pairs were cloned into recipient activated T cells or Jurkat cell line (TCR/CD3 Jurkat-luc cells (NFAT), Promega). Paired α and β chains were annotated based on bulk (that is, top TCR clonotypes obtained by TCR sequencing of tumor antigen FACS sorted TILs) or single-cell TCR sequencing data.

Autologous or HLA-matched allogeneic PBMCs were plated at 1 × 10^6^ cells per ml in p48-well plates in R8 medium supplemented with 50 IU ml^−1^ of IL-2 (Proleukin). T cells were activated with Dynabeads Human T Activator CD3/CD28 beads (Thermo Fisher Scientific) at a ratio of 0.75 beads:1 total PBMC. After 3 d of incubation at 37 °C and 5% CO_2_, beads were removed, and activated T cells were cultured for four extra days before electroporation or freezing.

For the transfection of TCRαβ pairs into T cells and Jurkat cells, the Neon electroporation system (Thermo Fisher Scientific) was used. Briefly, T cells and Jurkat cells were resuspended at 15–20 × 10^6^ cells per ml in buffer R (buffer from the Neon kit), mixed with 25–50 μg ml^−1^ of TCRα chain RNA together with 25–50 μg ml^−1^ of TCRβ chain RNA and electroporated with the following parameters: 1,600 V, 10 ms, three pulses and 1,325 V, 10 ms, three pulses, respectively. Electroporated cells were either incubated for 17–20 h at 37 °C or used immediately.

For the validation of antigen specificity, electroporated Jurkat cells were interrogated by pMHC-multimer staining with the following surface panel: anti-CD3, -CD4 (BioLegend), -CD8 (BD Biosciences), anti-mouse TCRβ-constant (Thermo Fisher Scientific) and Aqua viability dye (Thermo Fisher Scientific) (see the gating strategy in Supplementary Fig. 3a and Supplementary Methods). The following experimental controls were included: MOCK (transfection with PBS) and a control TCR (irrelevant cross-match of a TCRα and TCRβ chain) (Extended Data Fig. 7).

To assess anti-tumor reactivity of validated TCRs, 1 × 10^5^ TCR RNA-electroporated T cells and 3 × 10^4^ IFNγ-treated autologous tumor cells were co-cultured in IFNγ ELISpot assay. After 20–24 h of incubation, cells were recovered, and the upregulation of 4-1BB (CD137) was evaluated by staining with anti-4-1BB (Miltenyi Biotec), anti-CD3 (BioLegend), anti-CD4 and anti-CD8 (BD Biosciences), anti-mouse TCRβ-constant (Thermo Fisher Scientific) and viability dye Aqua (Thermo Fisher Scientific) (see the gating strategy in Supplementary Fig. 3b and Supplementary Methods). The following experimental controls of TCR transfection were included: MOCK (transfection with PBS), a control TCR (irrelevant cross-match of a TCRα and TCRβ chain) and, when available, a virus-specific TCR (Supplementary Fig. 4). Validation of tumor reactivity of TCRαβ pairs required (1) the background level of 4-1BB expression to be <20% in all control conditions; (2) the fold expansion of 4-1BB expression between transfected T cells exposed to autologous tumors and TCR T cells alone (background) to be >10; and (3) the percentage of 4-1BB expression after tumor challenge of transfected T cells and subtraction of the 4-1BB background obtained with transfected T cells alone to be >20% (Supplementary Fig. 4 and Extended Data Fig. 10b). Displayed data (Fig. 2e and Extended Data Fig. 10a) show the percentage of 4-1BB expression after tumor challenge of transfected T cells and subtraction of the 4-1BB background obtained with transfected T cells alone.

### Adoptive T cell transfer in immunodeficient IL-2 NOG mice

Tyr_508–514_-TCRα and TCRβ chains, divided by a Furin/GS linker/T2A element^51^, were cloned into a pCRRL-pGK lentiviral plasmid to produce high-titer replication-defective lentiviral particles, as previously described^52^. For primary human T cell transduction, CD8 T cells were negatively selected with beads (Miltenyi Biotec) from PBMCs of a healthy donor (apheresis filter from anonymous healthy donors following the legal Swiss guidelines under project P_123 with informed consent of the donors and with ethics approval from the Canton of Vaud (Lausanne)), activated and transduced as previously reported^52^, with minor modifications. Briefly, CD8 T cells were activated with anti-CD3/CD28 beads (Thermo Fisher Scientific) and added with lentiviral particles after overnight activation. Activation beads were removed after 5 d of T cell culture in R8 medium supplemented with IL-2 at 50 IU ml^−1^. At day 6, transduced T cells expressing the mouse TCRβ-constant region were sorted with a FACSAria III. Isolated Tyr_508–514_ TCR-transduced CD8 T cells were then expanded for 10 d in R8 medium and 50 IU ml^−1^ of IL-2 before mouse injection.

IL-2 NOG mice^34^ (Taconic Biosciences) were maintained in a conventional animal facility at the University of Lausanne under specific pathogen-free status. The housing conditions of mice were as follows: alternating cycles day/night of 12 h, humidity (55 ± 10%) and temperature (22 ± 1 °C). Six- to nine-week-old female mice were anesthetized with isoflurane and subcutaneously injected with 1 × 10^6^ tumor cells from melanoma patient 3. Once the tumors became palpable (at day 14), 5 × 10^6^ human Tyr_508–514_ TCR-transduced T cells were injected intravenously in the tail vein. Tumor volumes were measured by caliper twice a week and calculated as follows: volume = length × width × width/2. Mice were sacrificed by CO_2_ inhalation before the tumor volume exceeded 1,000 mm^3^ or when the state of the mice was affected over a certain threshold defined by a scoresheet taking into account physical and behavioral parameters. After mice were sacrificed, tumors were harvested and processed at the Tumor Processing Facility of the University of Lausanne. This study was approved by the Veterinary Authority of the Canton of Vaud (under license 3387) and performed in accordance with Swiss ethical guidelines.

### TCR-pMHC structure modeling

The three-dimensional structure of the three PHLPP2_N1186Y_-specific TCRs bound to peptide QSDNGLDSDY in complex with HLA-A*01:01 were modeled. Starting from V and J segment identifiers and from the CDR3 sequences, the full sequence of the constant and variable domains of TCRα and TCRβ were reconstituted based on IMGT/GENE-DB reference sequences^53^. Homology models of the TCR-pMHC complexes were generated using Rosetta v.3.10 (ref. ^54^) and Modeller v.9.21 (ref. ^55^). Template libraries include TCR, TCR-pMHC and pMHC structures retrieved from the Protein Data Bank^56^. The Rosetta ‘TCRmodel’ protocol^57^ was adapted to our approach and applied to find the respective templates and model TCRs (Supplementary Table 6). The orientation of modeled Vα and Vβ structure was performed based on Vα/Vβ templates, whereas the orientation of the TCR relative to the pMHC was performed based on TCR-pMHC templates, identified using sequence similarity (Supplementary Table 6). Side chains and backbones of the TCR-pMHC models were refined using Modeller^55^. A total of 1,500 models were produced for each TCR-pMHC. These models were subsequently ranked based on the discrete optimized potential energy as implemented in Modeller^55^. For each TCR-pMHC, the best model according to the score was selected for CDR loop refinement. The latter was performed by creating 100 alternative loop conformations using the kinematic closure loop modeling^58^ of Rosetta and subsequent refinement using the fast ‘relax’ protocol^54^. Molecular interactions were analyzed in the top five ranked models over the 1,600. The final TCR-pMHC structural model is the one with the highest number of favorable interactions within the top five high-score models. In these structure files, TCRα is chain D, TCRβ is chain E, peptide is chain C, MHC is chain A and β2-macroglobulin is chain B. Residue numbers start from 1 for each chain. Molecular graphics and analyses of the molecular interactions are presented, making use of the UCSF Chimera package^59^.

### Statistical analyses

Differences among averages of variables were compared using the one-tailed *t*-test for variables with normal distribution, as specified. Some variables underwent logarithmic transformation to obtain normality, as reported in the figure legends. Statistical analyses were performed using GraphPad Prism v.8.3.0.

### Reporting Summary

Further information on research design is available in the Nature Research Reporting Summary linked to this article.

## Online content

Any methods, additional references, Nature Research reporting summaries, source data, extended data, supplementary information, acknowledgements, peer review information; details of author contributions and competing interests; and statements of data and code availability are available at 10.1038/s41587-021-01072-6.

## Supplementary information

Supplementary Information Supplementary Figs. 1–4, Tables 1–6 and Methods.

Reporting Summary

## Data Availability

Exome and RNA sequencing data for patients 1, 2, 5, 6 and 7 have been uploaded to the European Genome-Phenome Archive (EGA) database under accession code EGAS00001005513. Data for patient 4 were deposited previously^45^ in the EGA database under accession codes EGAS00001003723 and EGAS00001003724. Data for patients 8 and 9 were deposited previously^15^ in the EGA database under accession code EGAS00001002803. The authors declare that additional data supporting the findings of this study are available in the article and its Supplementary Information. Other data are available from the corresponding authors upon reasonable request. The list of databases used throughout the study is as follows: ● IpMSDB database of hotspots of antigen presentation: 10.3389/fimmu.2017.01367 ● IMGT/GENE-DB reference sequence database: http://www.imgt.org/vquest/refseqh.html ● Protein Data Bank: https://www.rcsb.org/
