# Supplementary material for: Sensitive identification of neoantigens and cognate TCRs in human solid tumors
Source: Nat Biotechnol. 2021 Nov 15;40(5):656–60. doi: 10.1038/s41587-021-01072-6 (PMC9110298; doi:10.1038/s41587-021-01072-6)
Supplement: Supplementary file 1 — Supplementary Figs. 1–4, Tables 1–6 and Methods. [file 41587_2021_1072_MOESM1_ESM.pdf]

nature portfolio

https://doi.org/10.1038/s41587-021-01072-6

Supplementary information

Sensitive identification of neoantigens and cognate TCRs in human solid tumors

In the format provided by the authors and unedited

Supplementary Information for

Sensitive identification of neoantigens and cognate TCRs in human solid tumors

Marion Arnaud[1,2,3], Johanna Chiffelle[1,2,3], Raphaël Genolet[1,2,3], Blanca Navarro Rodrigo[1,2,3], Marta AS Perez[1,4], Florian Huber[1,2,3], Morgane Magnin[1,2,3,], Tu Nguyen-Ngoc[1,2,3], Philippe Guillaume[1,2,3], Petra Baumgaertner[1,2,3], Chloe Chong[1,2,3], Brian J. Stevenson[1,4], David Gfeller[1,3,4], Melita Irving[1,2,3], Daniel E. Speiser[1,2,3], Julien Schmidt[1,2,3], Vincent Zoete[1,4], Lana E. Kandalaft[1,2,3], Michal Bassani-Sternberg[1,2,3], Sara Bobisse[1,2,3], George Coukos[1,2,3,†,‡], Alexandre Harari[1,2,3,†,‡]

Affiliations:

[1]Ludwig Institute for Cancer Research, Lausanne Branch - University of Lausanne (UNIL), CH-1005, Switzerland

[2]Centre des Thérapies Expérimentales (CTE), Department of Oncology - Centre Hospitalier Universitaire Vaudois (CHUV), Lausanne CH-1011, Switzerland

[3]Department of Oncology - Centre Hospitalier Universitaire Vaudois (CHUV), Lausanne CH-1011, Switzerland

[4]SIB Swiss Institute of Bioinformatics, Lausanne CH-1015, Switzerland

†Equal contribution

‡Corresponding authors: George.coukos@chuv.ch & alexandre.harari@chuv.ch

Supplementary Figures

Supplementary Figure 1

a

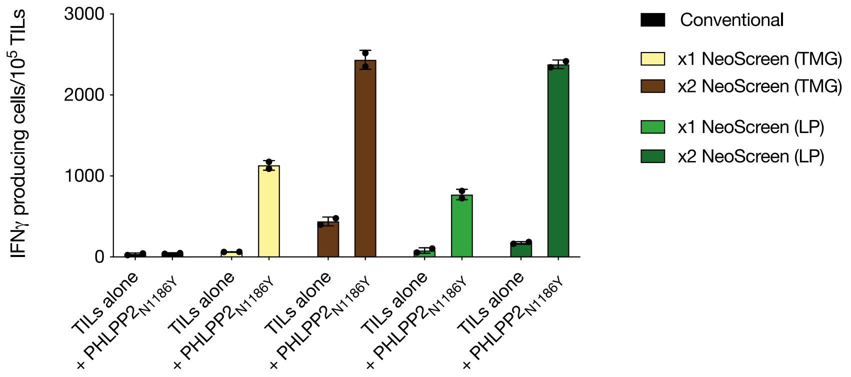

b

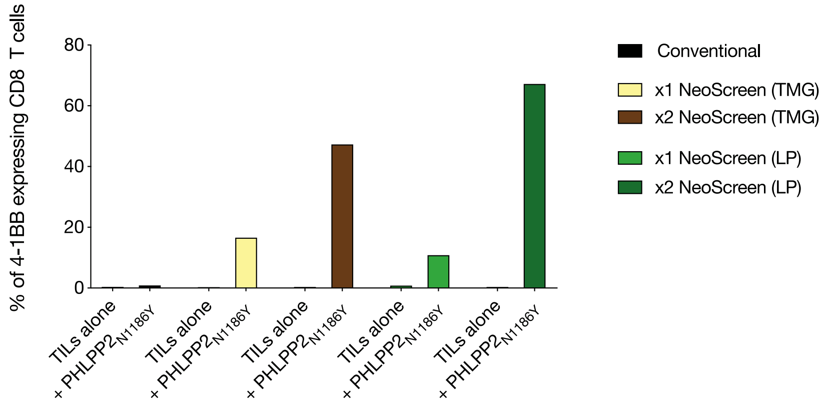

Supplementary Figure 1 | Evaluation of the frequency of tumor antigen-specific T cells by IFNγ ELISpot and 4-1BB upregulation. Example of T cell reactivity to neoepitope $PHLPP2_{N1186Y}$, assessed by IFNγ ELISpot assay (a) and 4-1BB upregulation (b). **a,** IFNγ Spot Forming Unit per 10^5 cells (mean±SD of duplicate) of TILs alone (negative control) or following stimulation with neoepitope $PHLPP2_{N1186Y}$. **b,** Frequency of neoepitope-specific CD8 T cells assessed by 4-1BB staining following cell recovery from ELISpot plates.

Supplementary Figure 2

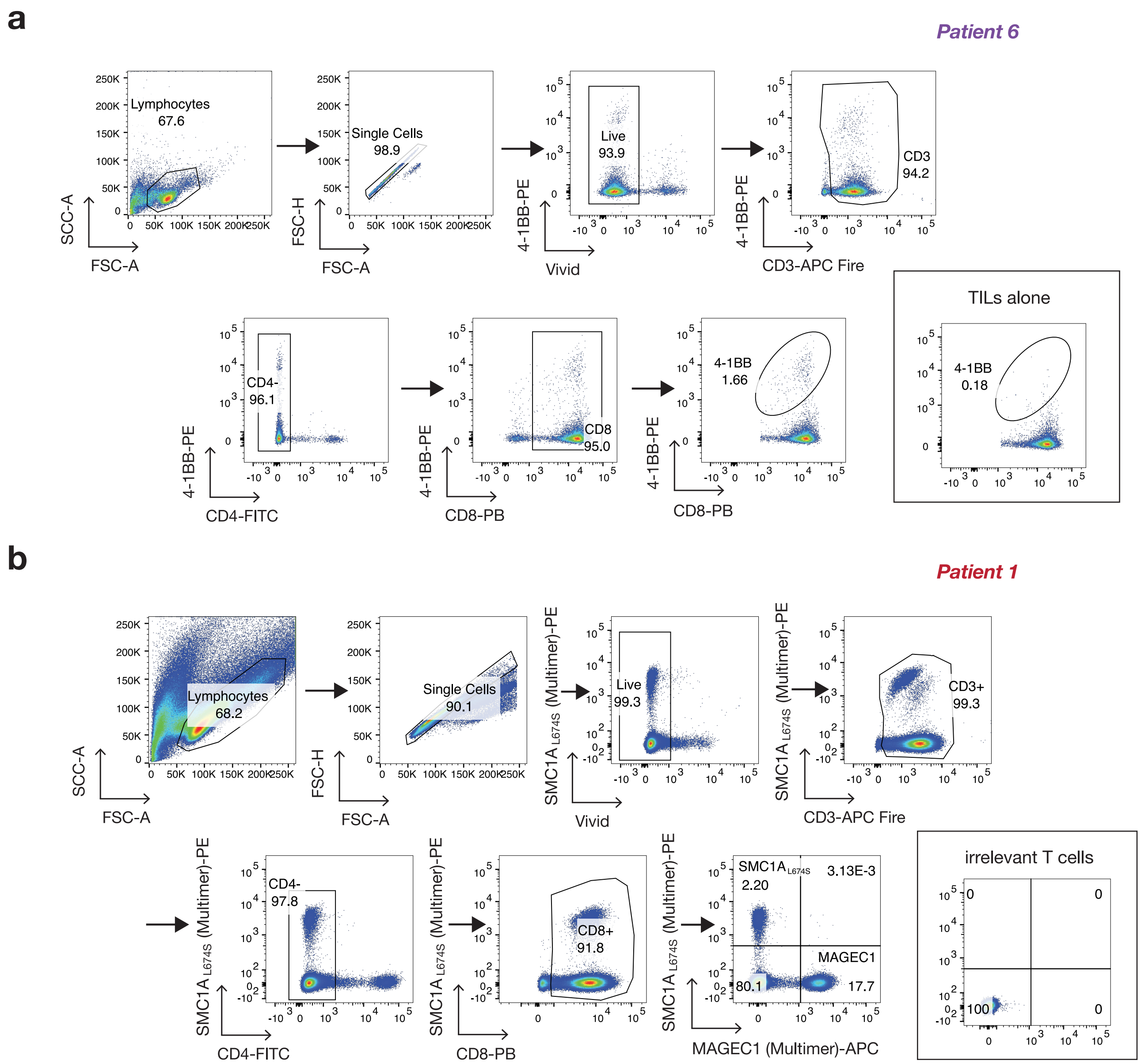

Supplementary Figure 2 | Evaluation of the frequency of tumor antigen-specific T cells by flow cytometry. a, Gating strategy and assessment of the frequency of tumor antigen-specific CD8 T cells by 4-1BB (CD137) staining following cell recovery from ELISpot plates; example of 4-1BB upregulation following stimulation with neoepitope $FCLR2_{R440M}$ of x2*NeoScreen* TILs from patient 6. **b,** Gating strategy and

assessment of the frequency of tumor antigen-specific CD8 T cells by pMHC multimer staining; example of MAGEC1 and $SMC1A_{L674S}$ -multimer staining of x2*NeoScreen* TILs from patient 1.

Supplementary Figure 3

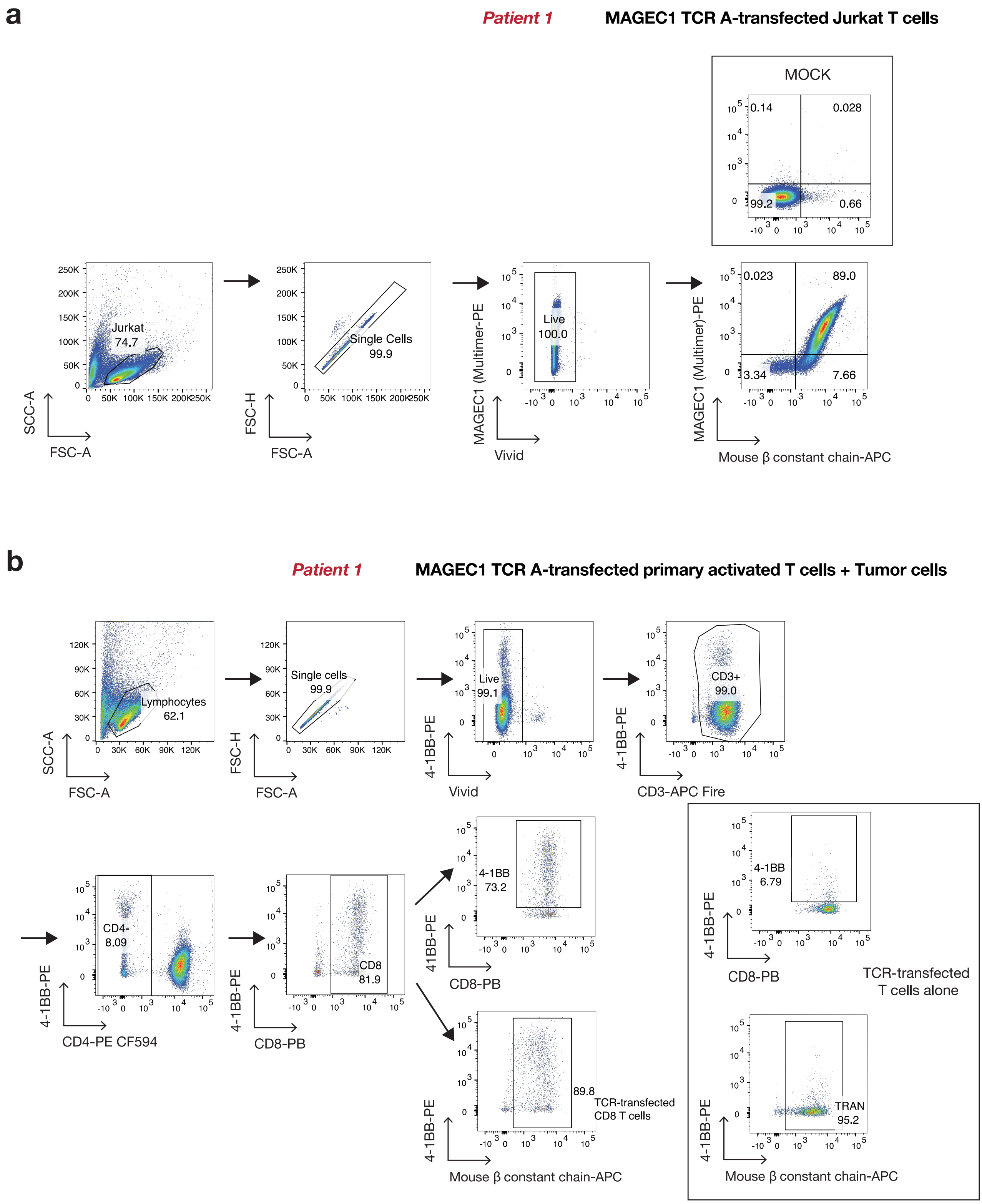

Supplementary Figure 3| Validation of antigen-specific TCRs and assessment of *in vitro* tumor recognition. a, Validation of antigen-specificity of MAGEC1 TCR A from patient 1 by transfection of Jurkat cells and pMHC multimer staining. **b,** Interrogation of *in vitro* tumor recognition of MAGEC1-specific TCR A by co-culture of TCR-transfected primary activated T cells with autologous tumor cells and evaluation of 4-1BB (CD137)-upregulation. (TRAN: control of transfection by evaluation of the expression of mouse β constant chain of transfected TCR).

Supplementary Figure 4

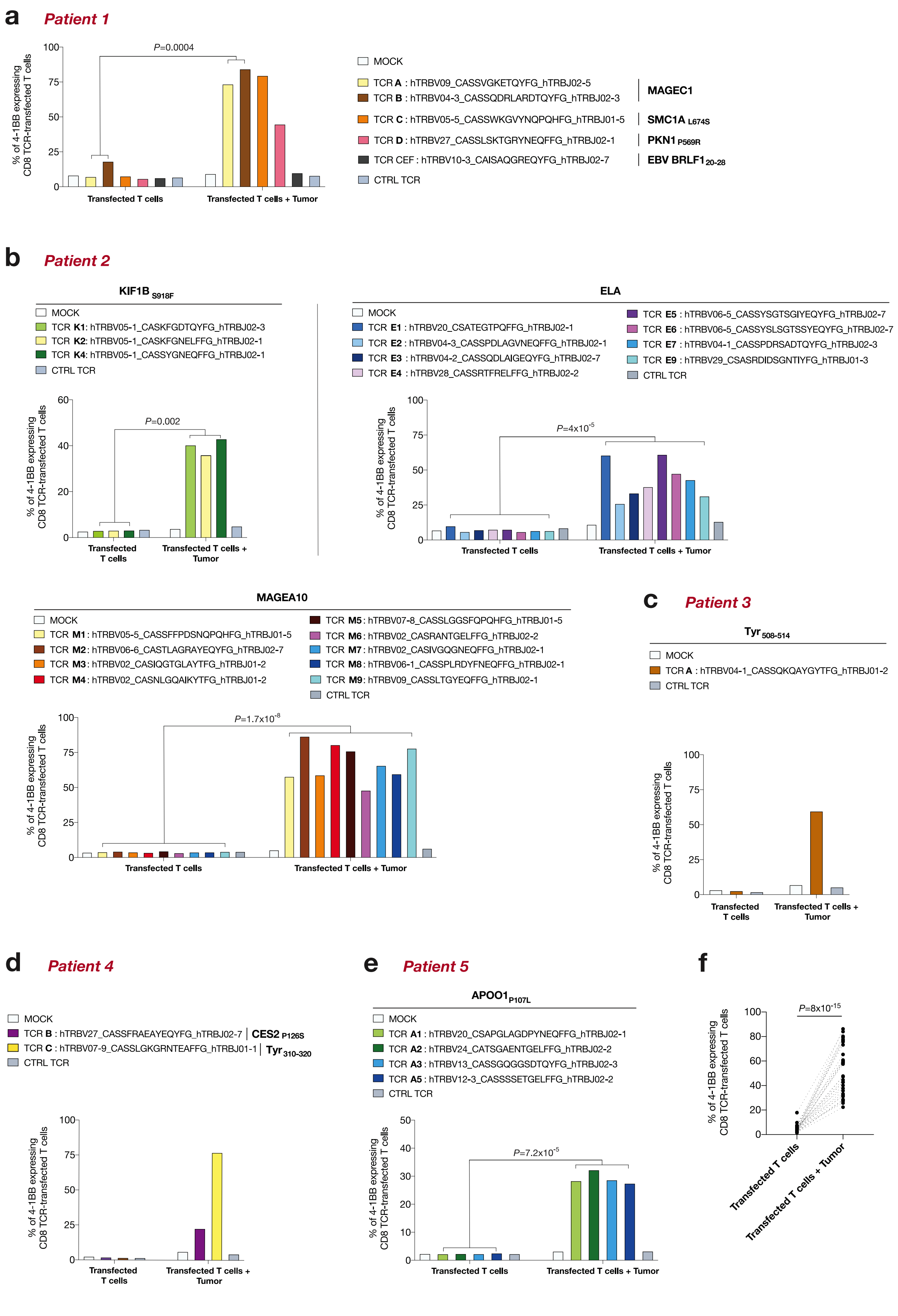

Supplementary Figure 4│ Validation of tumor reactivity of identified tumor antigen-specific TCRs. a-e, Overview of tumor reactivity of TCR-transfected CD8 T cells for patients 1-5. To assess antitumor reactivity of validated tumor antigen-specific TCRs (Table S5), autologous (patients 1-3 & 5) or HLA-matched (patient 4) TCR-transfected primary activated T cells were co-cultured with autologous tumor cells and 4-1BB up-regulation was measured. MOCK-T cells (transfected with PBS), T cells transfected with a control TCR (irrelevant crossmatch of a TCRα and β chain) and T cells transfected with a viral TCR (when available) were included as controls. The figure depicts the proportion of TCR-T cells expressing 4-1BB in presence or absence of tumor cells. The criteria for validation of tumor-reactive TCR$\alpha\beta$ pairs are detailed in the Methods section. Statistics were applied when $\geq$2 unique TCRs were targeting the same antigen and *P-values* were then determined with one-tailed paired t-tests. **f,** Cumulative statistics of all identified tumor antigen-specific TCRs (n=31). *P-value* was determined with a one-tailed paired t-test.

Supplementary Tables

Supplementary Table 1

Patient Id	Gender	Age	Tumor type	Stage at diagnosis	Samples's origin	Tumor sample	Other remarks
Patient 1	Male	44	Mucosal melanoma	pT4b pN0 M0, IIC	Lymph node metastasis	Tumor fragments	BRAF mutation
Patient 2	Male	60	Skin melanoma	pT3 pN1b M0, IIIB	Adrenal metastasis	Tumor fragments	BRAF mutation
Patient 3	Male	53	Melanoma of unknown origin	cT0 cN2b cM0, IIIB	Subcutaneous metastasis	Tumor fragments	BRAF mutation
Patient 4	Male	40	Skin melanoma	pT3b pN0 M0, IIC	subcutaneous metastasis	Tumor fragments	BRAF mutation
Patient 5	Female	65	Skin melanoma	pT3a pN1a cM0, IIIA	Lymph node metastasis	Tumor fragments	BRAF mutation
Patient 6	Male	69	Lung squamous cell carcinoma	pT2b pN0 M0, IIA	Lung	Tumor fragments	
Patient 7	Male	73	Colon adenocarcinoma	pT3 pN0 M0, II	caecum	Tumor fragments	MSI
Patient 8	Female	63	Epithelial ovarian adenocarcinoma	IV		Dissociated tumor cells	
Patient 9	Female	60	Epithelial ovarian adenocarcinoma	IV		Dissociated tumor cells	

Supplementary Table 1│ Description of patients. Patient identification number, gender, age, tumor type, stage at diagnosis, origin of sample, tumor sample type (tumor fragments or dissociated tumor cells) and further details (oncogene mutations, microsatellite instability (MSI)). Patients 8 and 9 were described previously[1].

Supplementary Table 2

Patient Id	Non synonymous mutations SNV*	HLA-A		HLA-B		HLA-C		Tumor antigen candidates †
Patient 1	46	01:01	03:01	27:05	57:01	01:02	06:02	83
Patient 2	95	02:05	32:01	07:02	44:03	04:01	07:02	124
Patient 3	71	02:01	01:01	08:01	40:01	07:01	03:04	139
Patient 4	128	01:01	23:01	07:02	15:01	14:02	12:03	191
Patient 5	70	02:01	26:01	44:02	51:08	05:01	05:01	70
Patient 8	46	02:01	29:02	44:02	44:04	05:01	16:01	19
Patient 9	10	23:01	23:01	14:02	50:01	06:02	08:02	1

Supplementary Table 2│ Number of SNVs, HLA class-I haplotype and number of tumor antigen candidates used for *NeoScreen* discovery. *single nucleotide variants (SNVs) related to tumor antigen candidates used for *NeoScreen* of patients 1 to 5; SNVs of patients 8 and 9 were already described[1]; **†** total number of tumor antigens used in *NeoScreen*; for patient 8, ten HLA-A*02:01-restricted epitopes detailed in Table S3 were also included.

Supplementary Table 3

Epitopes	HLA restriction	Peptide sequence
A2/hTERT (689-697)	A02:01	ILAKFLHWL
Survivin (96-104) (T98M)	A02:01	LTLGEFLKL
p53	A02:01	LLGRNSFE
Mesothelin (530-538)	A02:01	VLPLTVAEV
NY-ESO1 (457-165)	A02:01	SLLMWITQC
HER-2/neu (654-662)	A02:01	IISAVVGIL
HER-2/neu (369-377)	A02:01	KIFGSLAFL
HER-2/neu (689-697)	A02:01	RLLQETELV
WT-1 (126-134)	A02:01	RMFPNAPYL
MUC-1 (950-958)	A02:01	STAPPVHNV

Supplementary Table 3│ Description of shared HLA-A*02:01-restricted epitopes used for *NeoScreen* for patient 8. HLA-class I restriction and epitope sequence;

Supplementary Table 4

Patient Id	Gene	Non synonymous mutations SNVs	HLA restriction	Minimal epitope	Long epitope (for LP or TMG)
Patient 1	**SMC1A**	L674S	B27:05	RRWDEKAVDKSK	KAKARRWDEKAVDKSKEKKERL
	**MAGEC1▲**	NA	C01:02	FAFGEPREL	NA
	**PKN1**	P569R	A03:01	GTDSDSSRQK	ISVEKLNLGTDSDSSRQK
Patient 2	**ZNF397**	V223L	B44:03*	SEHESNLLW	NA
	**KIF1B**	S918F	A02:05	TADFDITEL	NA
	**DNAJC2**	C360R	B44:03*	QKLRNSRKTW	IKKERQKLRNSRKTWNHFSDN
	**MAGEA10✤**	NA	A02:05	GLYDGMEHL	NA
	**ELA✤**	NA	A02:05	ELAGIGILTV	NA
Patient 3	**Tyrosinase (508-514)▲**	NA	B08:01*	LPEEKQPL	NA
Patient 4	**NBEA•**	S2272L	B07:02	LPQARRILL	NA
	**Tyrosinase (310-320)▲•**	NA	B15:01	RLPSSADVEF	NA
	**CES2•**	P126S	B15:01	VQTFLGISF	NA
Patient 5	**APOO**	P107L	A02:01	ALPGFFPRL	WGLDSYDYLQNALPGFFPRLGVIG
	**ACTG1**	D24Y	B51:08*	AGYDAPRAV	GSGMCKAGFAGYDAPRAVFPSIVG
Patient 6	**NUP205**	Q471H	B35:03	EPLHTPTIM	HLELALEYWCPTEPLHTPTIMGSYLGVAHQR
	**FCRL2**	R440M	B35:03*	MPNPQEFTY	ISGESSATNEPRGASMPNPQEFTYSSPTPDM
	**KIT**	D165N	B35:03	IPNPKAGIM	GCQGKPLPKDLRFIPNPKAGIMIKSVKRAYH
Patient 7	**PHLPP2**	N1186Y	A01:01	QSDNGLDSDY	ATFSSNQSDNGLDSDYDQPVEGVITNGSKVE
Patient 8	**CDC20**	S231C	B44:02*	GEYISCVAWI	NA
	**Mesothelin (530-538)★**	NA	A02:01*	VLPLTVAEV	NA
	**WT-1 (126-134)★**	NA	A02:01*	RMFPNAPYL	NA
	**HER-2/neu (369-377)★**	NA	A02:01*	KIFGSLAFL	NA
Patient 9	**HS6ST1**	S405I	A23:01*	DYMIHIIEKW	NA

Supplementary Table 4 | Validated tumor-associated antigens and neoepitopes. Patient identification number; gene (✤ MAGEA10 & ELA were considered since patient 2 was previously vaccinated with these TAAs; ▲ TAAs were identified as described in the Methods section; • patient 4 had a total number of 191 antigen candidates and thus they were split into two pools for *in vitro* TIL expansion, $CES2_{P126S}$ was in a first pool, noted *NeoScreen* (1), and $NBEA_{S2272L}$ and $tyrosinase_{310-320}$ were in the second pool *NeoScreen* (2) (Methods); ★ patient 8 was interrogated with a list of TAAs, as described in Table S3; single nucleotide variants (SNVs) (NA: not applicable); HLA restriction (predicted HLA-restrictions not confirmed by pMHC multimers are shown with an *); minimal peptide sequence; long peptide sequence

(when tandem minigenes (TMG) and/or long peptides (LP) were used at the initiation of TIL cultures).

Supplementary Table 5

Patient Id	Gene	Non synonymous mutations SNVs	HLA restriction	Minimal epitope	TCR Id	TCR pMHC beta chain	TCR pMHC alpha chain	Antigen validation TCR cloning	Tumor reactivity (4-1BB upregulation)
Patient 1	MAGEC1		C01:02	FAFGEPREL	A	hTRBV09_CASSVGKETQYFG_hTRBJ02-5	hTRAV13-1_CAASDSSASKIIFG_hTRAJ03	Yes	66%
					B	hTRBV04-3_CASSQDRLARDTQYFG_hTRBJ02-3	hTRAV14_CAMREPYYNQGGKLIFG_hTRAJ23	Yes	66%
	SMC1A	L674S	B27:05	RRWDEKAVDKSK	C	hTRBV05-5_CASSWKGVYNQPQHFG_hTRBJ01-5	hTRAV26-2_CILRAVYVRFG_hTRAJ43	Yes	72%
	PKN1	P569R	A03:01	GTDSDSSRQK	D	hTRBV27_CASSLSKTGRYNEQFFG_hTRBJ02-1	hTRAV20_CAVQATSGSARQLTFG_hTRAJ22	Yes	39%
Patient 2	MAGEA10		A02:05	GLYDGMEHL	M1	hTRBV05-5_CASSFFPDSNQPQHFG_hTRBJ01-5	hTRAV17_CATVFYGNNRLAFG_hTRAJ07	Yes	54%
					M2	hTRBV06-6_CASTLAGRAYEQYFG_hTRBJ02-7	hTRAV12-1_CVHMEYGNKLVFG_hTRAJ47	Yes	82%
					M3	hTRBV02_CASIQGTGLAYTFG_hTRBJ01-2	hTRAV22_CAVGVLRDYKLSFG_hTRAJ20	Yes	55%
					M4	hTRBV02_CASNLGQAIKYTFG_hTRBJ01-2	hTRAV21_CAVAVFPGNQFYFG_hTRAJ49	Yes	77%
					M5	hTRBV07-8_CASSLGGSFQPQHFG_hTRBJ01-5	hTRAV38-2_CAYRSAMYSGGGADGLTFG_hTRAJ45	Yes	71%
					M6	hTRBV02_CASRANTGELFFG_hTRBJ02-2	hTRAV17_CATDAYNFNKFYFG_hTRAJ21	Yes	45%
					M7	hTRBV02_CASIVGQGNEQFFG_hTRBJ02-1	hTRAV19_CALSERPGGATNKLIFG_hTRAJ32	Yes	62%
					M8	hTRBV06-1_CASSPLRDYFNEQFFG_hTRBJ02-1	hTRAV36_CAVCDSWGKLQFG_hTRAJ24	Yes	56%
					M9	hTRBV09_CASSLTGYEQFFG_hTRBJ02-1	hTRAV12-2_CAVKGSGTSYGKLTFG_hTRAJ52	Yes	74%
					M10	hTRBV11-3_CASSQVSPSYNEQFFG_hTRBJ02-1	NA	NA	NA
	Melan-A (ELA)	A27L	A02:05	ELAGIGILTV	E1	hTRBV20_CSATEGTPQFFG_hTRBJ02-1	hTRAV12-2_CAVNSGGGADGLTFG_hTRAJ45	Yes	51%
					E2	hTRBV04-3_CASSPDLAGVNEQFFG_hTRBJ02-1	hTRAV27_CAGEAFGGNYQLIWGAG_hTRAJ33	Yes	20%
					E3	hTRBV04-2_CASSQDLAIGEQYFG_hTRBJ02-7	hTRAV35_CAGPNAGGTSYGKLTFG_hTRAJ52	Yes	26%
					E4	hTRBV28_CASSRTFRELFFG_hTRBJ02-2	hTRAV26-1_CIVRVPSDNFNKFYFG_hTRAJ21	Yes	30%
					E5	hTRBV06-5_CASSYSGTSGIYEQYFG_hTRBJ02-7	hTRAV19_CALSEARGGADGLTFG_hTRAJ45	Yes	54%
					E6	hTRBV06-5_CASSYSLSGTSSYEQYFG_hTRBJ02-7	hTRAV12-1_CVVNGGYNNNDMRFG_hTRAJ43	Yes	42%
					E7	hTRBV04-1_CASSPDRSADTQYFG_hTRBJ02-3	hTRAV12-2_CAVNAGNQFYFG_hTRAJ49	Yes	36%
					E8	hTRBV04-1_CASSQDFSSGNEQFFG_hTRBJ02-1	NA	NA	NA
					E9	hTRBV29_CSASRDIDSGNTIYFG_hTRBJ01-3	hTRAV12-2_CAVGDYKLSFG_hTRAJ20	Yes	25%
	KIF1B	S918F	A02:05	TADFDITEL	K1	hTRBV05-1_CASKFGDTQYFG_hTRBJ02-3	hTRAV20_CAVQAPYSGAGSYQLTFG_hTRAJ28	Yes	37%
					K2	hTRBV05-1_CASKFGNELFFG_hTRBJ02-1	hTRAV01-2_CAVIRGYSGAGSYQLTFG_hTRAJ28	Yes	33%
					K3	hTRBV09_CASSVVGTREQFFG_hTRBJ02-1	NA	NA	NA
					K4	hTRBV05-1_CASSYGNEQFFG_hTRBJ02-1	hTRAV01-2_CAVRTGYSGAGSYQLTFG_hTRAJ28	Yes	40%
Patient 3	Tyrosinase (508-514)		B08:01*	LPEEKQP	A	hTRBV04-1_CASSQKQAYGYTFG_hTRBJ01-2	hTRAV21_CAVSPNYGQNFVFG_hTRAJ26	Yes	57%
Patient 4	NBEA	S2272L	B07:02	LPQARRILL	A	hTRBV04-1_CASSQDWAGGSTDTQYFG_hTRBJ02-3	NA	NA	NA
	CES2	P126S	B15:01	VQTFLGISF	B	hTRBV27_CASSFRAEAYEQYFG_hTRBJ02-7	hTRAV26-2_CITTNAGKSTFG_hTRAJ27	Yes	21%
	Tyrosinase (310-320)		B15:01	RLPSSADVEF	C	hTRBV07-9_CASSLGKGRNTEAFFG_hTRBJ01-1	hTRAV01-2_CAVRDNNNDMRFG_hTRAJ43	Yes	75%
Patinet 5	APOO	P107L	A02:01	ALPGFFPRL	A1	hTRBV20_CSAPGLAGDPYNEQFFG_hTRBJ02-1	hTRAV12-2_CAVGGSARQLTFG_hTRAJ22	Yes	26%
					A2	hTRBV24_CATSGAENTGELFFG_hTRBJ02-2	hTRAV12-2_CAVNPLNNFNKFYFG_hTRAJ21	Yes	30%
					A3	hTRBV13_CASSGQGGSDTQYFG_hTRBJ02-3	hTRAV20_CAVRVAGGTSYGKLTFG_hTRAJ52	Yes	26%
					A4	hTRBV07-6_CASSLNGEDEQFFG_hTRBJ02-1	NA	NA	NA
					A5	hTRBV12-3_CASSSSETGELFFG_hTRBJ02-2	hTRAV20_CAVQGGSNYKLTFG_hTRAJ53	Yes	25%
					A6	hTRBV27_CASSSMENTGELFFG_hTRBJ02-2	NA	NA	NA
					A7	hTRBV07-2_CASSLYGPEETQYFG_hTRBJ02-5	NA	NA	NA
Patient 6	NUP205	Q471H	B35:03	EPLHTPTIM	A	hTRBV04-3_CASSQGTGAYEQYFG_hTRBJ02-7	hTRAV12-1_CVADTGRRALTFG_hTRAJ05	Yes	Tumor cell line NA
					B	hTRBV05-1_CASSLVTGGLVDYEQYFG_hTRBJ02-7	hTRAV27_CAGAGSNSGYALNFG_hTRAJ41	Yes	
					C	hTRBV07-6_CASSLLTGTGELFFG_hTRBJ02-2	hTRAV12-1_CVVNEGMRFG_hTRAJ43	Yes	
					D	hTRBV28_CASSSLAGDYEQYFG_hTRBJ02-7	hTRAV12-1_CVGDLNGQNFVFG_hTRAJ26	Yes	
					E	hTRBV07-2_CASSFTSGHEQYFG_hTRBJ02-7	hTRAV12-1_CVVNYGGSQGNLIFG_hTRAJ42	Yes	
	KIT	D165N	B35:03	IPNPKAGIM	F	hTRBV02_CASEGLTYNEQFFG_hTRBJ02-1	NA	NA	
					G	hTRBV02_CASEGLAGAEQYFG_hTRBJ02-7	hTRAV08-1_CAVNSGGGADGLTFG_hTRAJ45	Yes	
					H	hTRBV02_CASSEGFGTSDSGDTQYFG_hTRBJ02-3	NA	NA	
					I	hTRBV05-4_CASSFFTGISYNEQFFG_hTRBJ02-1	NA	NA	
Patient 7	PHLPP2	N1186Y	A01:01	QSDNGLDSDY	A	hTRBV10-3_CAISGGSVGEQYFG_hTRBJ02-7	hTRAV23_CAAPMPMDTGRRALTFG_ hTRAJ05	Yes	Tumor cell line NA
					B	hTRBV05-4_CASTLSTGQGIYGYTFG_hTRBJ01-2	hTRAV21_CAVSSGSARQLTFG_hTRAJ22	Yes	
					C	hTRBV05-4_CASSPTTSGRIGELFFG_hTRBJ02-2	hTRAV21_CAVGGSGSARQLTFG_hTRAJ22	Yes	

Supplementary Table 5| Description of tumor antigen-specific TCRs. Patient identification number (Id); gene; single nucleotide variants (SNVs); HLA restriction (predicted restrictions not confirmed by pMHC multimers are shown with an *); peptide sequence; TCR Id; validated TCRα and TCRβ chains when TCR$\alpha\beta$ pair was validated by TCR cloning as described in the Methods section (NA: not applicable when the TCRα chain was not identified by TCR cloning); Percentage of 4-1BB expression after tumor challenge of transfected T cells, after subtraction of the 4-1BB background obtained with transfected T cells alone (NA: when autologous tumor cell lines were not available).

Supplementary Table 6

Patient Id	Gene	Non synonymous mutations SNVs	HLA restriction	Minimal epitope	TCR Id	TCR pMHC beta chain	TCR pMHC alpha chain	Templates TCRα || TCRβ || a:b orientation || peptide-MHC || TCR:p:MHC orientation
Patient 7	**PHLPP2**	N1186Y	A01:01	QSDNGLDSDY	**A**	hTRBV10-3_CAISGGSVGEQYFG_hTRBJ02-7	hTRAV23_CAAPMPMDTGRRALTFG_ hTRAJ05	3mff, 3c6l, 3vxm, 1uh3 || 3qeq, 3vxm|| 1u3h:1u3h|| 5brs, 5bs0, 6at9 || 5brs, 5bs0
					**B**	hTRBV05-4_CASTLSTGQGIYGYTFG_hTRBJ01-2	hTRAV21_CAVSSGSARQLTFG_hTRAJ22	6eh4, 4eup|| 6bj2, 4h1l || 6eh4:6eh4|| 5brs, 5bs0, 6at9 || 5brs, 5bs0
					**C**	hTRBV05-4_CASSPTTSGRIGELFFG_hTRBJ02-2	hTRAV21_CAVGGSGSARQLTFG_hTRAJ22	6eh4, 4h1l || 6bj2, 4h1l || 6eh4:6eh4|| 5brs, 5bs0, 6at9 || 5brs, 5bs0

Supplementary Table 6| Protein Data Bank entries used to model the 3D structures of the of PHLPP2$_{N1186Y}$- specific TCRs A, B and C. Patient identification number (Id); gene; single nucleotide variants (SNVs); HLA restriction; peptide sequence; TCR Id; validated TCRα and TCRβ chains; Modelled structures of three PHLPP2$_{N1186Y}$- specific TCRs; Protein Data Bank entries used as templates to model the 3D structures are displayed on Fig. 2d and Extended Data Fig. 8.

Reference

1. Bobisse, S. *et al.* Sensitive and frequent identification of high avidity neo-

epitope specific CD8+ T cells in immunotherapy-naive ovarian cancer. *Nat. Commun.* **9**, 1092 (2018).

Supplementary Methods

Marker	Fluorochrome	Clone	Source	Concentration (amount per 100uL)	Cat nb	Lot nb
Aqua	Excitation 405nm	NA	Thermo Fischer Scientific	0.3	L34966	2157201
Calcein	Excitation 488nm	NA	Thermo Fischer Scientific	0.1	C3099	2098542
CD3	APC Fire 750	SK7	Biolegend	1	344840	B286176
CD4	FITC	SK3	Biolegend	1	344604	B244280
CD4	PE-CF594	RPA-T4	BD Biosciences	3	562281	9186815
CD45	APC	HI30	Biolegend	5	304012	B272156
CD8α	PB	RPA-T8	BD Biosciences	3	558207	9294848
CD19	BV711	SJ25C1	BD Biosciences	3	563036	8337862
CD19	PE-Cy7	SJ25C1	BD Biosciences	4	557835	9287460
CD40	PE DAZZLE 594	5C3	Biolegend	1	334342	B242793
CD70	FITC	Ki-24	BD Biosciences	8	555834	7159745
CD80	V450	L307.4	BD Biosciences	1	560444	6266951
CD83	APC	HB15e	Biolegend	10	305312	B260800
CD86	PE	IT2.2	Biolegend	2	305406	B210795
CD137 (41BB)	PE	4B4-1	Miltenyi	2	130-093-475	5201008496
CD137-L (41BB-L)	PE-Vio 770	REA254	Miltenyi	5	130-118-976	5180403066
DAPI	Excitation 358nm	NA	Thermo Fischer Scientific	2x10^(-5)	D3571	1890543
HLA ABC	PerCPCy5.5	W6/32	Biolegend	2	311420	B227388
HLA DR	BV605	L243	Biolegend	2	307640	B215412
Mouse TCR ß constant	APC	H57-597	Thermo Fischer Scientific	4	17-5961-81	2142290
OX40-L	PE	ik-1	BD Biosciences	1.5	558164	9087756

Flow cytometry analysis: markers, fluorochromes, clones, sources, concentrations, category numbers (Cat nb) and lot numbers of the antibodies used for flow cytometry.
