# Supplementary material for: Sensitive identification of neoantigens and cognate TCRs in human solid tumors
Source: Nat Biotechnol. 2021 Nov 15;40(5):656–60. doi: 10.1038/s41587-021-01072-6 (PMC9110298; doi:10.1038/s41587-021-01072-6)
Supplement: Supplementary file 2 — Reporting Summary [file 41587_2021_1072_MOESM2_ESM.pdf]

nature research

Corresponding author(s): Alexandre Harari and George Coukos

Last updated by author(s): Aug 4, 2021

Reporting Summary

Nature Research wishes to improve the reproducibility of the work that we publish. This form provides structure for consistency and transparency in reporting. For further information on Nature Research policies, see our Editorial Policies and the Editorial Policy Checklist.

Statistics

For all statistical analyses, confirm that the following items are present in the figure legend, table legend, main text, or Methods section.

n/a	Confirmed	
☐	☒	The exact sample size (*n*) for each experimental group/condition, given as a discrete number and unit of measurement
☐	☒	A statement on whether measurements were taken from distinct samples or whether the same sample was measured repeatedly
☐	☒	The statistical test(s) used AND whether they are one- or two-sided<br>*Only common tests should be described solely by name; describe more complex techniques in the Methods section.*
☒	☐	A description of all covariates tested
☐	☒	A description of any assumptions or corrections, such as tests of normality and adjustment for multiple comparisons
☐	☒	A full description of the statistical parameters including central tendency (e.g. means) or other basic estimates (e.g. regression coefficient) AND variation (e.g. standard deviation) or associated estimates of uncertainty (e.g. confidence intervals)
☐	☒	For null hypothesis testing, the test statistic (e.g. *F*, *t*, *r*) with confidence intervals, effect sizes, degrees of freedom and *P* value noted<br>*Give P values as exact values whenever suitable.*
☒	☐	For Bayesian analysis, information on the choice of priors and Markov chain Monte Carlo settings
☒	☐	For hierarchical and complex designs, identification of the appropriate level for tests and full reporting of outcomes
☒	☐	Estimates of effect sizes (e.g. Cohen's *d*, Pearson's *r*), indicating how they were calculated

Our web collection on statistics for biologists contains articles on many of the points above.

Software and code

Policy information about availability of computer code

Data collection

For sequencing, software used are publicly available and stated in the Methods section where applicable.
For sequencing: bcl2fastq Conversion Software (v. 1.84, Illumina); bcl2fastq2w Conversion Software (v.2.20, Illumina)
For HLA-typing: MiniSeq instrument (Illumina)
For flow cytometry: BD Fortessa
For FACS sorting: BD FACS ARIA II, BD FACS Melody
For single-cell TCR sequencing: 10x Genomics

Data analysis

For graphical analyses: Graph Pad Prism v8.3.0.;

For sequencing and MS analyses, software used are publicly available and stated in the Methods section where applicable.
- Exome analyses: NeoDisc v1.2, GenomeAnalysisTK (GATK) v3.7, Picard Tools v2.9.0;
- HLA typing: Assign TruSight software v2.1;
- RNA sequencing analyses: RNA-Star v2.4.2a, Cufflinks v2.2.1, GTEx v7;
- MS analyses: MaxQuant v1.5.9.4i, Comet 2017.01 rev. 2, Apache Spark cluster computing framework;
- HLA-peptide binding prediction: netMHC v3.4, netMHCpan-3.0, PRIME v1.0, MixMHCpred v2;
- scRNAseq analyses: Cell Ranger v.3.0.1, Seurat v3;
- General: RStudio v3.5.1 and Python v3.6, STRING-db v11, GTEx v7, ComplexHeatmap v1.99.4, SSRCalc
vQ.0;
- NewAnce: The NewAnce code is available on the following GitHub link: https://github.com/bassanilab/NewAnce.git;

For flow cytometry: FACS DIVA Software v9.0, FlowJo X;
For single-cell TCR sequencing: Cell Ranger v3.1.0, 10x Genomics
For TCR pMHC modelling: Rosetta version v3.10 (https://www.rosettacommons.org/software), Modeller v9.21 (https://salilab.org/modeller/)

and UCSF Chimera v1.11.2 (https://www.cgl.ucsf.edu/chimera/);

For manuscripts utilizing custom algorithms or software that are central to the research but not yet described in published literature, software must be made available to editors and reviewers. We strongly encourage code deposition in a community repository (e.g. GitHub). See the Nature Research guidelines for submitting code & software for further information.

Data

Policy information about availability of data

All manuscripts must include a data availability statement. This statement should provide the following information, where applicable:

- Accession codes, unique identifiers, or web links for publicly available datasets
- A list of figures that have associated raw data
- A description of any restrictions on data availability

Exome and RNA sequencing data for patients 1, 2, 5, 6 and 7 have been uploaded to the European Genome-phenome Archive (EGA) database under the accession code EGAS00001005513. Data for patient 4 was deposited previously in EGA database under the accession codes EGAS00001003723 and EGAS00001003724. Data for patient 8 and 9 were deposited previously in EGA database under the accession code EGAS00001002803.
The list of databases used throughout the study is the following:
- IpMSDB database of hotspots of antigen presentation: https://doi.org/10.3389/fimmu.2017.01367
- IMGT/GENE-DB reference sequence database: http://www.imgt.org/vquest/refseqh.html
- Protein Data Bank: https://www.rcsb.org/
The authors declare that additional data supporting the findings of this study are available within the article and its Supplementary Information. Other data are available from the corresponding authors upon reasonable request.

Field-specific reporting

Please select the one below that is the best fit for your research. If you are not sure, read the appropriate sections before making your selection.

☒ Life sciences ☐ Behavioural & social sciences ☐ Ecological, evolutionary & environmental sciences

For a reference copy of the document with all sections, see nature.com/documents/nr-reporting-summary-flat.pdf

Life sciences study design

All studies must disclose on these points even when the disclosure is negative.

Sample size	For the in vitro studies, number of healthy donors and/or technical replicates were chosen according to the complexity of the assay and for the expected biological variability. All in vitro studies for patient samples were performed according to sample availability (e.g. tumor samples).<br>For the in vivo studies, 5 millions TCR-transduced T cells/mouse were used. We achieved a sample size of minimum 5 animals per treatment group according to the expected biological variability, which was estimated to be sufficient to reproducibly observe statistically significant differences. The estimation was based on calculations via the following website: https://www.openepi.com/SampleSize/SSMean.htm.
Data exclusions	No data were excluded from analyses.
Replication	All attempts at replication were successful. For antigen screening of TIL cultures, IFNg Enzyme-Linked ImmunoSpot (ELISpot) and pMHC-multimer complexes staining were performed at the end of cultures and antigens were validated by ≥3 independent experiments.
Randomization	For the in vitro antigen screening of TILs, randomization is not applicable. For interrogation and validation of antigen and tumor reactivity of TCR, autologous patient samples were used, except in one case where we selected an allogenic sample based on HLA-match.<br>Tumor burden was evaluated by calipering the same day of T cell transfer. No mice were excluded at any point. Following tumor burden measure, mice were randomly assigned into treatment and control groups (TCR-transduced & untransduced T cells) such that each group had the same overall average tumor volume. Apheresis filters were obtained from anonymous donors.
Blinding	For in vitro experiments, including antigen screening of TILs and interrogation of antigen and tumor reactivity of TCRs, the blinding concept is not applicable.<br>For in vivo experiments, caliper measurements and data analyses were performed in a non-blinded fashion. A complete blinding was not achievable because it would have required additional operators. The operator of in vivo assays was aware which were the untransduced versus the TCR-transduced T cells.

Reporting for specific materials, systems and methods

We require information from authors about some types of materials, experimental systems and methods used in many studies. Here, indicate whether each material, system or method listed is relevant to your study. If you are not sure if a list item applies to your research, read the appropriate section before selecting a response.

Materials & experimental systems

n/a	Involved in the study
☐	☒ Antibodies
☐	☒ Eukaryotic cell lines
☒	☐ Palaeontology and archaeology
☐	☒ Animals and other organisms
☐	☒ Human research participants
☒	☐ Clinical data
☒	☐ Dual use research of concern

Methods

n/a	Involved in the study
☒	☐ ChIP-seq
☐	☒ Flow cytometry
☒	☐ MRI-based neuroimaging

Antibodies

Antibodies used	Antibodies were titrated for optimal staining. Aqua live Dye BV510 (L34966, Thermo Fisher Scientific, Lot no 2157201) was used to assess viability. The following fluorophore conjugated antibodies were used for phenotypic analysis of CD40-activated B cells: PE-Cy7 mouse anti-human CD19 (clone SJ25C1, cat 557835, lot n° 9287460, BD Biosciences), V450 mouse anti-human CD80 (clone L307.4, cat 560444, lot n° 6266951, BD Biosciences), FITC mouse anti-human CD70 (clone Ki-24, cat 555834, lot n° 7159745, BD Biosciences), PerCPCy5.5 mouse anti-human HLA-ABC (clone W6/32, cat 311420, lot n° B227388, Biolegend), BV605 mouse anti-human HLA-DR (clone L243, cat 307640, lot n° B215412, Biolegend), APC mouse anti-human CD83 (clone HB15e, cat 305312, lot n° B260800, Biolegend), PE mouse anti-human CD86 (clone IT2.2, cat 305406, lot n° B210795, Biolegend) PE DAZZLE 594 mouse anti-human CD40 (clone 5C3, cat 334342, lot n° B242793, Biolegend) (Panel 1),and BV711 mouse anti-human CD19 (clone SJ25C1, cat 563036, lot n° 8337862, BD Biosciences), PE mouse anti-human OX40L (clone ik-1, cat 558164, lot n° 9087756, BD Biosciences), PE-Vio 770 mouse anti-human 4-1BBL (clone REA254, cat 130-118-976, lot n° 5180403066, Miltenyi) (Panel 2). The following fluorophore conjugated antibodies were used for antigen screening of TIL cultures by 4-1BB upregulation or pMHC multimer staining: PE mouse anti-human 4-1BB (clone 4B4-1, cat 130-093-475, lot n° 5201008496, Miltenyi) or PE and or APC-conjugated pMHC multimers (in house production) together with APC-Fire 750 mouse anti-human CD3 (clone SK7, cat 344840, lot n° B286176, Biolegend), FITC mouse anti-human CD4 (clone SK7, cat 344604, lot n° B244280, Biolegend), PB mouse anti-human CD8a (clone RPA-T8, cat 558207, lot n° 9294848, BD Biosciences). For the purification of antigen-specific T cells, the same antibodies as just mentioned were used, with the exception of the mouse anti-human CD3, which was not used for FACS sorting. For single-cell TCR sequencing, dissociated tumor samples were stained with APC mouse anti-human CD45 (clone HI30, cat 304012, lot n°272156, Biolegend) and viability dyes: Calcein-AM (C3099, Thermo Fisher Scientific, Lot no 2098542) and DAPI (D3571, Thermo Fisher Scientific, Lot no 2157201) and viable CD45 cells were FACS purified. For the validation of antigen-specific TCRs by TCR cloning, the following panel was used: APC-Fire 750 mouse anti-human CD3 (clone SK7, cat 344840, lot n° B286176, Biolegend), PB mouse anti-human CD8a (clone RPA-T8, cat 558207, lot n° 9294848, BD Biosciences), PE-CF594 mouse anti-human CD4 (clone RPA-T4, cat 562281, lot n° 9186815, BD Biosciences), APC hamster anti-mouse TCRb constant (clone H57-597, cat 17-5961-81, lot n° 2142290) together with PE mouse anti-human 4-1BB (clone 4B4-1, cat 130-093-475, lot n° 5201008496, Miltenyi) (if reactivity assessed by up-regulation of 4-1BB) or PE-conjugated pMHC multimers (in house production, if reactivity assessed by pMHC-multimer). To assess the in vitro anti-tumor reactivity of validated antigen-specific TCR, the latter panel with the anti-human 4-1BB was used.
Validation	Antibodies' concentration validation was empirically determined in the lab. All primary antibodies were validated and titrated with human TILs, PBMCs or additional irrelevant cells that were either activated or resting, depending on each antibody. All titrations are provided in a Supplementary Method Table included in the Supplementary Information. pMHC Multimers were produced in house and were validated in vitro using TILs encompassing the relevant antigen reactivity (previously validated by IFNg ELISpot).

Eukaryotic cell lines

Policy information about cell lines

Cell line source(s)	TCR/CD3 Jurkat Cells (NFAT) from Promega (cat J131A, Promega Academic Access Program).
Authentication	The cell lines were not authenticated.
Mycoplasma contamination	All cell lines were routinely tested for mycoplasma contamination and found negative.
Commonly misidentified lines (See ICLAC register)	No cell lines from the ICLAC register were used.

Animals and other organisms

Policy information about studies involving animals; ARRIVE guidelines recommended for reporting animal research

Laboratory animals	IL-2 NOG mice were obtained from Taconic Biosciences and maintained in a conventional animal facility at the University of Lausanne under specific pathogen–free status. The housing conditions of mice were the following: alternating cycles day/night of 12hours, humidity (55%, +/-10%), temperature (22°C, +/- 1°C). Six- to nine-week old female mice were used in this study.
Wild animals	No wild animals were used in this study.
Field-collected samples	No field collected samples were used in this study.

Ethics oversight	This study was approved by the Veterinary Authority of the Canton de Vaud (under the license VD3387) and performed in accordance with Swiss ethical guidelines.

Note that full information on the approval of the study protocol must also be provided in the manuscript.

Human research participants

Policy information about studies involving human research participants

Population characteristics	Buffy coats and apheresis filters from anonymous healthy donors were collected from the local transfusion center. Patients included stage III/IV metastatic melanoma, ovarian, non-small cell lung cancer and colorectal cancer patients. Please see Supplementary Table 1 in Supplementary information for details about the population characteristics. Samples from four melanoma patients enrolled in a phase I clinical trial of TIL ACT were collected at baseline (NCT03475134).
Recruitment	For healthy donors: recruitment is not applicable because it is performed by the local blood transfusion centre, Lausanne, Switzerland;<br><br>Patients were enrolled under protocols approved by the respective institutional regulatory committees at the University of Pennsylvania, USA, and Lausanne university hospital (Ethics Committee, University Hospital of Lausanne-CHUV), Switzerland.
Ethics oversight	For patients' samples: Ethics Committee, University Hospital of Lausanne-CHUV & the regulatory committee of the University of Pennsylvania; All patients signed informed consents.<br><br>For healthy donors: collection following the legal Swiss guidelines under the project P_123 with informed consent of the donors and with Ethics Approval from the Canton of Vaud (Lausanne).

Note that full information on the approval of the study protocol must also be provided in the manuscript.

Flow Cytometry

Plots

Confirm that:

☒ The axis labels state the marker and fluorochrome used (e.g. CD4-FITC).

☒ The axis scales are clearly visible. Include numbers along axes only for bottom left plot of group (a 'group' is an analysis of identical markers).

☒ All plots are contour plots with outliers or pseudocolor plots.

☒ A numerical value for number of cells or percentage (with statistics) is provided.

Methodology

Sample preparation	For pMHC-multimer staining, cells were washed once and resuspended in FACS buffer containing pMHC multimer(s). Cells were incubated at 4 degrees for 45 minutes and washed once before cell surface staining. For cell surface staining preparation, cells were washed once and resuspended in PBS containing LIVE/DEAD dye and the antibody cocktail. Cells were incubated at 4 degrees for 20 minutes and washed twice before acquisition. Cells were not fixed prior to acquisition.<br><br>Antigen-specific CD8 T cells were FACS sorted using either in-house pMHC multimers or based on 4-1BB (CD137) up-regulation. The staining process was similar to the above mentioned one and cells were additionally filtered before sorting. Purified antigen-specific cells underwent TCR sequencing analyses immediately after sorting.<br><br>To assess the efficacy in vivo of antigen-specific TCR, CD8 T cells were activated with anti-CD3/CD28 beads and added with lentiviral particles after overnight activation. After 6 days, transduced T cells expressing the mouse TCRbeta-constant region were stained with antibody and LIVE/DEAD dye and sorted by FACS. Isolated TCR-transduced CD8 T cells were then expanded for 10 days in R8 medium and 50IU/mL IL-2 before mouse injection.<br><br>For downstream single-cell TCR analyses, dissociated tumor samples were filtered and resuspended in PBS + 1% Gelatin + 0.1% RNasin and cells were stained first with viability dye Calcein AM for 15min at room temperature (RT) and next with anti-CD45 at 4 degrees for 20 minutes. Cells were then resuspended in PBS complemented with 0.04% BSA + 0.1%RNasin, next DAPI staining was performed and finally CD45 live cells were purified by FACS.
Instrument	BD Fortessa; BD FACS Melody ; BD FACS ARIA II; Beckman Coulter FACS Astrios;
Software	Collection: FACS DIVA<br>Analysis: FlowJo X
Cell population abundance	Due to the low numbers (<200'000 cells) of purified antigen-specific CD8 T cells, population abundance was not assessed post sorting and cells were immediately processed for TCR sequencing analysis. FACS-purified cells were counted using Trypan blue and viability was found to be >90%.
Gating strategy	Starting cell population was gated on a linear SSC-A/FSC-A plot. Single cells were discriminated on a linear FSC-H or FSC-W/

Gating strategy	FSC-A plot. Live cells were determined by exclusion from positive Live/Dead stained cells. Positive/Negative populations were determined with negative controls, as detailed in the Supplementary Information.

☒ Tick this box to confirm that a figure exemplifying the gating strategy is provided in the Supplementary Information.
